# Quantum Nonlocality in Any Forked Tree-Shaped Network

**DOI:** 10.3390/e24050691

**Published:** 2022-05-13

**Authors:** Lihua Yang, Xiaofei Qi, Jinchuan Hou

**Affiliations:** 1 School of Mathematical Science, Shanxi University, Taiyuan 030006, China; 201912211008@email.sxu.edu.cn; 2 School of Mathematics and Information Technology, Yuncheng University, Yuncheng 044000, China; 3 College of Mathematics, Taiyuan University of Technology, Taiyuan 030024, China; houjinchuan@tyut.edu.cn

**Keywords:** quantum correlation, nonlocality, Bell inequality, quantum network

## Abstract

In the last decade, much attention has been focused on examining the nonlocality of various quantum networks, which are fundamental for long-distance quantum communications. In this paper, we consider the nonlocality of any forked tree-shaped network, where each node, respectively, shares arbitrary number of bipartite sources with other nodes in the next “layer”. The Bell-type inequalities for such quantum networks are obtained, which are, respectively, satisfied by all $\left(t_{n}-1\right)$-local correlations and all local correlations, where $t_{n}$ denotes the total number of nodes in the network. The maximal quantum violations of these inequalities and the robustness to noise in these networks are also discussed. Our network can be seen as a generalization of some known quantum networks.

## 1. Introduction

Quantum correlation is one of the main characteristics that distinguishes quantum mechanics from classical mechanics. In the last few decades, quantum nonlocality has been studied extensively both in theory [1,2,3] and experiment [4,5,6]. It is found that quantum nonlocality is a powerful resource in quantum information science, such as secure cryptography [7,8], quantum key distribution [9], randomness certification [10], and distributed computing [11]. Bell inequalities are often used to detect quantum nonlocality [12,13,14]. Violations of Bell inequalities imply the existence of nonlocal correlations.

Different from the usual Bell nonlocality, where entanglement is distributed from one common source, the multi-locality in quantum networks features several independent sources. By performing joint measurements, this leads to stronger correlations throughout the whole network [15], which is fundamental for long-distance quantum communications. Nonlocality of correlations generated in such networks was first observed in a bilocal network [16,17,18]. Later, the authors in [18] obtained the bilocal inequalities for bilocal networks, and the scholars in [19] explicitly examined quantum violations of the bilocal inequalities for pure states and mixed states, respectively. Since then, the nonlocality of various quantum networks were explored, including chain-shaped networks [20], star-shaped networks [21,22,23], triangle networks [24], and other networks in [25,26,27,28,29,30,31,32]. Furthermore, stronger forms of network nonlocality were examined in [33,34,35].

The tree-tensor networks are also important quantum networks. They have wide applications, such as in quantum simulations [36,37,38,39], entanglement transitions [40], and quantum-assisted machine learning [41]. Recently, nonlocal correlations of a special class of tree-tensor networks, so-called “two-forked” tree-shaped networks were studied in [42]. In this network, there are $\left(2^{n}-1\right)$ parties (nodes) distributed in *n* “layers” (n≥2), where each layer *k* (1≤k≤n) has $2^{k-1}$ parties, and each party in the layer *k* shares a source with another party in the layer k−1 and with other two parties in the layer k+1. Thus, this network is a $\left(2^{n}-1\right)$-partite system with $\left(2^{n}-2\right)$ independent sources.

The purpose of the present paper is to consider the nonlocality of any forked tree-shaped network. In this tree-shaped network, $t_{n}$ parties are arranged in an *n* “layer” scenario (n≥2), and the (k,j) party in the layer *k*, respectively, shares a source with another party in the layer k−1 and with other $l_{kj}-l_{k(j-1)}$ parties in the layer k+1 (2≤k≤n−1, j≥1), where $l_{k0}=0$ and $l_{kj}-l_{k(j-1)}$ is an arbitrary positive integer. Denote the total number of parties in layer *k* by $p_{k}$(k=1,2,⋯,n), satisfying $p_{1}=1$. Write $t_{n}=p_{1}+\cdots +p_{n}$. Thus, the whole network is a $t_{n}$-partite system with $\left(t_{n}-1\right)$ independent sources. In particular, if $l_{kj}-l_{k(j-1)}=2$ for all (k,j), this tree-shaped network reduces to the network in [42].

The rest of this paper is organized as follows. In Section 2, we discuss any forked tree-shaped network with $t_{n}$ parties and $\left(t_{n}-1\right)$ independent sources. We explicitly examine the nonlocality of the network for the case of n=3 and generalize the results to arbitrary n≥3. Moreover, the $\left(t_{n}-1\right)$-local inequalities of the networks and quantum violations of the corresponding inequalities for pure states and mixed states are obtained. Besides, we also compare this network with some known quantum network scenarios. Some conclusions are presented in Section 3. The detailed proofs of the main results are provided in Appendix A.

## 2. Nonlocality in Any Forked Tree-Shaped Network Scenario

**Figure 1:**
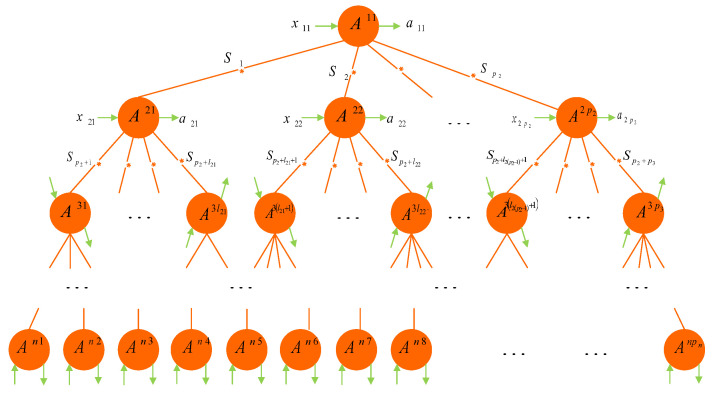
The general any forked tree-shaped network consists of $t_{n}$ parties (A${}^{11}$, A${}^{21}$, ⋯, A${}^{2p_{2}}$, A${}^{31}$, ⋯, A${}^{3p_{3}}$,⋯, A${}^{n1}$, ⋯, A${}^{np_{n}}$), and $t_{n}-1$ independent sources $S_{1},\cdots ,S_{t_{n}-1}$. Denote by $x_{i}$ and $a_{i}$ the input and output of each party A${}^{i}$ (*i* = 11, 21, ⋯, $np_{n}$), respectively.

In this section, we consider the nonlocality of a general tree-shaped network; see Figure 1.

This general tree-shaped network has *n* “layers” (n≥2), where each layer *k* has $p_{k}$ parties (nodes) with $p_{1}=1$, say Alice k1 (A${}^{k1}$), ⋯, Alice $kp_{k}$ (A${}^{kp_{k}}$), 1≤k≤n; each party A${}^{kj}$ in the layer *k* shares one source with another party in the layer k−1 and with $l_{kj}-l_{k(j-1)}$ parties in the layer k+1, where $l_{kj}-l_{k(j-1)}$ is an arbitrary positive integer, except that $l_{11}=p_{2}>1$, $1\leq j\leq p_{k}$, 2≤k≤n−1, and $l_{k0}=0$. It is clear that $l_{kp_{k}}=p_{k+1}$, k=1,2,⋯,n−1. Write $t_{n}=p_{1}+p_{2}+\cdots +p_{n}$. Thus, this general tree-shaped quantum network concerns a $t_{n}$-partite system with $t_{n}-1$ independent sources. In addition, the $t_{n}-1$ independent sources $S_{1},\cdots ,S_{t_{n}-1}$ are characterized by independent hidden variables $\lambda_{1},\cdots ,\lambda_{t_{n}-1}$, respectively. Denote by $x_{i}$ and $a_{i}$ the input and output of party A${}^{i}$ ($i=11,21,\cdots ,np_{n}$), respectively.

We say that the correlations in the tree-shaped network of Figure 1 are local if the joint probability distribution satisfies

(1) $$\begin{array}{cc} & P(a_{11},a_{21},\cdots ,a_{\left(n-1\right)p_{n-1}},a_{n1},\cdots ,a_{np_{n}}|x_{11},x_{21},\cdots ,x_{\left(n-1\right)p_{n-1}},x_{n1},\cdots ,x_{np_{n}})\\ = & \int \cdots \int P\left(\lambda_{1},\cdots ,\lambda_{t_{n}-1}\right)[P\left(a_{11}|x_{11},\lambda_{1},\cdots ,\lambda_{p_{2}}\right)P\left(a_{21}|x_{21},\lambda_{1},\lambda_{p_{2}+1},\cdots ,\lambda_{p_{2}+l_{21}}\right)\\ & \cdots P(a_{\left(n-1\right)p_{n-1}}|x_{\left(n-1\right)p_{n-1}},\lambda_{t_{n-1}-1},\lambda_{t_{n-1}+l_{\left(n-1\right)\left(p_{n-1}-1\right)}},\cdots ,\lambda_{t_{n}-1})\\ & \cdot P\left(a_{n1}|x_{n1},\lambda_{t_{n-1}}\right)\cdots P\left(a_{np_{n}}|x_{np_{n}},\lambda_{t_{n}-1}\right)]d\lambda_{1}\cdots d\lambda_{t_{n}-1}; \end{array}\;$$

and moreover, if $P(\lambda_{1},\cdots ,\lambda_{t_{n}-1})$ in Equation (1) can be decomposed into

(2) $$P\left(\lambda_{1},\cdots ,\lambda_{t_{n}-1}\right)=P_{1}\left(\lambda_{1}\right)\cdots P_{t_{n}-1}\left(\lambda_{t_{n}-1}\right)\;\mathrm{with}\;\int P_{i}\left(\lambda_{i}\right)d\lambda_{i}=1,\;\;i=1,2,\cdots ,t_{n}-1,\;$$

then we say that the correlations in the tree-shaped network of Figure 1 are $\left(t_{n}-1\right)$-local. Under the source independence restriction Equation (2), correlations that cannot be decomposed into Equation (1) are said to be non-$\left(t_{n}-1\right)$-local.

### 2.1. $\left(t_{3}-1\right)$-Local Network Scenario

**Figure 2:**
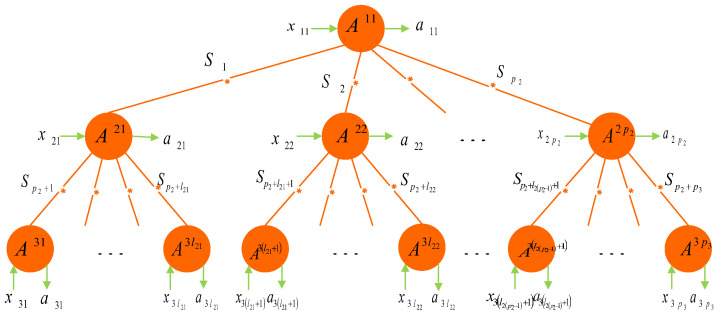
For the case of n=3, the any forked tree-shaped network consists of $t_{3}$ parties (A${}^{11}$, A${}^{21}$, ⋯, A${}^{2p_{2}}$, A${}^{31}$, ⋯, A${}^{3p_{3}}$) and $\left(t_{3}-1\right)$ independent sources $S_{1},\cdots ,S_{t_{3}-1}$. Let $x_{11},x_{21},\cdots ,x_{2p_{2}},x_{31},\cdots ,x_{3p_{3}}$ and $a_{11},a_{21},\cdots ,a_{2p_{2}},a_{31},\cdots ,a_{3p_{3}}$ be the corresponding input and output of each party, respectively.

If n=3 in Figure 1, then it reduces to the network of Figure 2.

The network of Figure 2 is a $t_{3}$-partite system with $t_{3}-1$ independent sources, where party A${}^{11}$ shares $p_{2}$ sources with parties A${}^{21}$, A${}^{22}$, ⋯, A${}^{2p_{2}}$; party A${}^{2j}$ shares $l_{2j}-l_{2(j-1)}+1$ sources with parties A${}^{11}$, A${}^{3(l_{2(j-1)}+1)}$, ⋯, A${}^{3l_{2j}}$, where $j=1,2,\cdots ,p_{2}$ and $l_{20}=0$. Let $l_{2p_{2}}=p_{3}$, and then, $t_{3}=1+p_{2}+p_{3}$.

**Figure 3:**
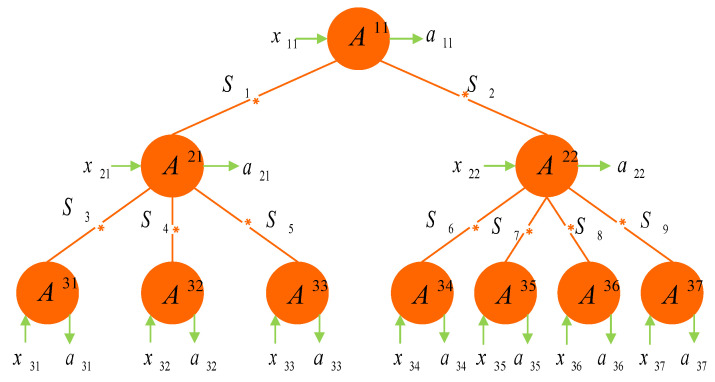
A tree-shaped network involves 10 parties, A${}^{11}$, A${}^{21}$, A${}^{22}$, A${}^{31}$, ⋯, A${}^{37}$, and 9 sources, $S_{1}$, ⋯, $S_{9}$. Denote by $x_{11}$, $x_{21}$, $x_{22}$, $x_{31}$, ⋯, $x_{37}$ and $a_{11}$, $a_{21}$, $a_{22}$, $a_{31}$, ⋯, $a_{37}$ the input and output of each party, respectively. Here, $l_{11}=2$, $l_{21}=3$, $l_{22}=7$ and $p_{2}=2$, $p_{3}=7$, $t_{1}=1$, $t_{2}=3$, $t_{3}=10$.

To illustrate Figure 2, we give a concrete example. Let $p_{2}=2$, $p_{3}=l_{22}=7$, and $l_{21}=3$. Then, we obtain the network of Figure 3, which is a 10-partite system with nine independent sources.

For the case n=3, the correlations obtained in the network of Figure 2 are called local if the probability distribution can be decomposed as

(3) $$\begin{array}{cc} & P(a_{11},a_{21},\cdots ,a_{2p_{2}},a_{31},\cdots ,a_{3p_{3}}|x_{11},x_{21},\cdots ,x_{2p_{2}},x_{31},\cdots ,x_{3p_{3}})\\ = & \int \cdots \int d\lambda_{1}\cdots d\lambda_{t_{3}-1}P\left(\lambda_{1},\cdots ,\lambda_{t_{3}-1}\right)[P\left(a_{11}|x_{11},\lambda_{1},\cdots ,\lambda_{p_{2}}\right)\\ & \cdot P\left(a_{21}|x_{21},\lambda_{1},\lambda_{p_{2}+1},\cdots ,\lambda_{p_{2}+l_{21}}\right)\cdots P\left(a_{2p_{2}}|x_{2p_{2}},\lambda_{p_{2}},\lambda_{p_{2}+l_{2(p_{2}-1)}+1},\cdots ,\lambda_{t_{3}-1}\right)\\ & \cdot P\left(a_{31}|x_{31},\lambda_{p_{2}+1}\right)\cdots P\left(a_{3p_{3}}|x_{3p_{3}},\lambda_{t_{3}-1}\right)], \end{array}\;$$

and are called $\left(t_{3}-1\right)$-local if they have a decomposition form of Equation (3) with the additional restriction

(4) $$P\left(\lambda_{1},\lambda_{2},\cdots ,\lambda_{t_{3}-1}\right)=P_{1}\left(\lambda_{1}\right)P_{2}\left(\lambda_{2}\right)\cdots P_{t_{3}-1}\left(\lambda_{t_{3}-1}\right).\;$$

Here, the output of every party depends on the corresponding input and all connected sources. Correlations that do not meet Equations (3) and (4) are said to be non-$\left(t_{3}-1\right)$-local.

#### 2.1.1. $\left(t_{3}-1\right)$-Locality Inequality

In what follows, we consider the case that each party A${}^{i}$$\left(i=11,21,\cdots ,3p_{3}\right)$ has binary input $x_{i}\left(\in \left\{0,1\right\}\right)$ with binary output $a_{i}\left(\in \left\{0,1\right\}\right)$, respectively. We develop inequalities that are fulfilled by all probability distributions satisfying Equations (3) and (4), but which may be violated by measuring quantum states distributed in the tree-shaped network of Figure 2.

**Theorem** **1.** *Any $\left(t_{3}-1\right)$-local correlation in the tree-shaped network of Figure 2 must satisfy the following inequalities:*

(5) $$|I_{i_{1},\cdots ,i_{t_{2}},0}{|}^{\frac{1}{p_{3}}}+{\left|I_{j_{1},\cdots ,j_{t_{2}},1}\right|}^{\frac{1}{p_{3}}}\leq 1,\;\;\forall i_{1},\cdots ,i_{t_{2}},j_{1},\cdots ,j_{t_{2}}\in \left\{0,1\right\},\;$$

*where*

$$I_{i_{1}\left(j_{1}\right),\cdots ,i_{t_{2}}\left(j_{t_{2}}\right),k}=\frac{1}{2^{p_{3}}}\sum_{x_{31},\cdots ,x_{3p_{3}}}{\left(-1\right)}^{kl}\langle A_{i_{1}\left(j_{1}\right)}^{11}A_{i_{2}\left(j_{2}\right)}^{21}\cdots A_{i_{t_{2}}\left(j_{t_{2}}\right)}^{2p_{2}}A_{x_{31}}^{31}\cdots A_{x_{3p_{3}}}^{3p_{3}}\rangle ,$$

$$\begin{array}{cc} & \langle A_{x_{11}}^{11}A_{x_{21}}^{21}\cdots A_{x_{2p_{2}}}^{2p_{2}}A_{x_{31}}^{31}\cdots A_{x_{3p_{3}}}^{3p_{3}}\rangle \\ = & \sum_{\begin{array}{c} a_{11},a_{21},\cdots ,a_{2p_{2}},\\ a_{31},\cdots ,a_{3p_{3}} \end{array}}{\left(-1\right)}^{m}P\left(a_{11},a_{21},\cdots ,a_{2p_{2}},a_{31},\cdots ,a_{3p_{3}}|x_{11},x_{21},\cdots ,x_{2p_{2}},x_{31},\cdots ,x_{3p_{3}}\right), \end{array}$$

*k∈{0,1}, $l=x_{31}+\cdots +x_{3p_{3}}$, $m=a_{11}+a_{21}+\cdots +a_{2p_{2}}+a_{31}+\cdots +a_{3p_{3}}$, $A_{x_{11}}^{11}$ denotes the observable for binary inputs $x_{11}$ of party $A^{11}$, and $A_{x_{21}}^{21}$, ⋯, $A_{x_{2p_{2}}}^{2p_{2}}$, $A_{x_{31}}^{31}$, ⋯, $A_{x_{3p_{3}}}^{3p_{3}}$ are similarly defined.*

Note that the subscript $t_{2}$ in Ineqs. (5) indicates the total number of parties $A^{11}$, $A^{21}$, ⋯, $A^{2p_{2}}$. By Theorem 1, we see that violation of Ineqs. (5) for at least one possible $(i_{1},\cdots ,i_{t_{2}},j_{1}$, $\cdots ,j_{t_{2}})$ guaranteeing the non-$\left(t_{3}-1\right)$-local nature of the correlations generated by the network of Figure 2. Besides, each of the above $2^{2t_{2}}$ inequalities is tight.

To see this, we give an explicit $\left(t_{3}-1\right)$-local decomposition, which is able to saturate the bound. Consider the following strategy:

$$P\left(a_{11}|x_{11},\lambda_{1},\cdots ,\lambda_{p_{2}}\right)=\left\{\begin{array}{c} 1,\;\mathrm{if}\;a_{11}=\lambda_{1}⊕\cdots ⊕\lambda_{p_{2}},\\ 0,\;\mathrm{else}, \end{array}\right.$$

$$P\left(a_{21}|x_{21},\lambda_{1},\lambda_{p_{2}+1},\cdots ,\lambda_{p_{2}+l_{21}}\right)=\left\{\begin{array}{c} 1,\;\mathrm{if}\;a_{21}=\left(\lambda_{1}⊕\lambda_{p_{2}+1}⊕\cdots ⊕\lambda_{p_{2}+l_{21}-1}\right)\lambda_{p_{2}+l_{21}},\\ 0,\;\mathrm{else}, \end{array}\right.$$

⋯⋯

$$\begin{array}{cc} & P(a_{2p_{2}}|x_{2p_{2}},\lambda_{p_{2}},\lambda_{p_{2}+l_{2(p_{2}-1)}+1},\cdots ,\lambda_{t_{3}-1})\\ = & \left\{\begin{array}{c} 1,\;\mathrm{if}\;a_{2p_{2}}=\left(\lambda_{p_{2}}⊕\lambda_{p_{2}+l_{2(p_{2}-1)}+1}⊕\cdots ⊕\lambda_{t_{3}-2}\right)\lambda_{t_{3}-1},\\ 0,\;\mathrm{else}, \end{array}\right. \end{array}$$

$$P\left(a_{3j}|x_{3j},\lambda_{p_{2}+j},\tau_{3j}\right)=\left\{\begin{array}{c} 1,\;\mathrm{if}\;a_{3j}=\lambda_{p_{2}+j}⊕\tau_{3j}x_{3j},\\ 0,\;\mathrm{else}, \end{array}\right.\;\forall j=1,2,\cdots ,p_{3}.$$

Here, $\lambda_{m}$ are hidden variables of shared sources $S_{m}$ with $P_{m}\left(\lambda_{m}=0\right)=1$ ($m=1,2,\cdots ,t_{3}-1)$, and $\tau_{3j}$ are sources of local randomness for party A${}^{3j}$ with $P_{j}\left(\tau_{3j}=0\right)=r$ and $P_{j}\left(\tau_{3j}=1\right)=1-r$, r∈[0,1] ($j=1,2,\cdots ,p_{3}$). A simple calculation gives $I_{i_{1},\cdots ,i_{t_{2}},0}=r^{p_{3}}$ and $I_{j_{1},\cdots ,j_{t_{2}},1}={\left(1-r\right)}^{p_{3}}$ for any $i_{1},\cdots ,i_{t_{2}},j_{1},\cdots ,j_{t_{2}}\in \left\{0,1\right\}$. Hence, $|I_{i_{1},\cdots ,i_{t_{2}},0}{|}^{\frac{1}{p_{3}}}+{\left|I_{j_{1},\cdots ,j_{t_{2}},1}\right|}^{\frac{1}{p_{3}}}=1$, reaching the bound for all $i_{1},\cdots ,i_{t_{2}},j_{1},\cdots ,j_{t_{2}}\in \left\{0,1\right\}$.

As for the nonlocality correlations in the network of Figure 2, we give a set of Bell-type inequalities.

**Theorem** **2.** *Every local correlation in the tree-shaped network of Figure 2 satisfies the following inequalities:*

(6) $$|I_{i_{1},\cdots ,i_{t_{2}},0}\left|+\right|I_{j_{1},\cdots ,j_{t_{2}},1}|\leq 1,\;\;\forall i_{1},\cdots ,i_{t_{2}},j_{1},\cdots ,j_{t_{2}}\in \left\{0,1\right\},\;$$

*where $I_{i_{1},\cdots ,i_{t_{2}},0}$ and $I_{j_{1},\cdots ,j_{t_{2}},1}$ are defined as in Theorem 1.*

By Theorem 2, the violation of at least one of the $2^{2t_{2}}$ Ineqs.(6) guarantees that the corresponding correlations generated by the network are nonlocal. Apparently, the set of $\left(t_{3}-1\right)$-local correlations is a subset of the set of local correlations in the network of Figure 2.

For the proofs of Theorems 1 and 2, see Appendix A.

#### 2.1.2. Quantum Violations of $\left(t_{3}-1\right)$-Local Inequalities

Now, we consider the network of Figure 2 involving $\left(t_{3}-1\right)$ independent quantum sources, each generating a bipartite quantum state. Then, the overall quantum state of this network has the form

$$\rho =\rho_{A_{1}^{11}A_{1}^{21}}⊗\cdots ⊗\rho_{A_{p_{2}}^{11}A_{1}^{2p_{2}}}⊗\rho_{A_{2}^{21}A^{31}}⊗\cdots ⊗\rho_{A_{l_{21}+1}^{21}A^{3l_{21}}}⊗\cdots ⊗\rho_{A_{l_{2p_{2}}-l_{2(p_{2}-1)}+1}^{2p_{2}}A^{3p_{3}}}$$

with state space $H=H_{A^{11}}⊗H_{A^{21}}⊗\cdots ⊗H_{A^{2p_{2}}}⊗H_{A^{31}}⊗\cdots ⊗H_{A^{3p_{3}}}$, where $H_{A^{11}}=H_{A_{1}^{11}}⊗\cdots ⊗H_{A_{p_{2}}^{11}}$ and $H_{A^{2i}}=H_{A_{1}^{2i}}⊗\cdots ⊗H_{A_{l_{2i}-l_{2(i-1)}+1}^{2i}}$, $i=1,\cdots ,p_{2}$. For simplicity, we write

$$\rho =\rho_{A^{11}A^{21}}⊗\cdots ⊗\rho_{A^{11}A^{2p_{2}}}⊗\rho_{A^{21}A^{31}}⊗\cdots ⊗\rho_{A^{21}A^{3l_{21}}}⊗\cdots ⊗\rho_{A^{2p_{2}}A^{3p_{3}}}.$$

Once each party receives particles from its all-connecting sources, it performs suitable measurement. The resulting joint probability distribution has the form

$$\begin{array}{cc} & P(a_{11},a_{21},\cdots ,a_{2p_{2}},a_{31},\cdots ,a_{3p_{3}}|x_{11},x_{21},\cdots ,x_{2p_{2}},x_{31},\cdots ,x_{3p_{3}})\\ = & \mathrm{tr}[\left(M_{a_{11}|x_{11}}⊗M_{a_{21}|x_{21}}⊗\cdots ⊗M_{a_{3p_{3}}|x_{3p_{3}}}\right)\left(\rho_{A^{11}A^{21}}⊗\rho_{A^{11}A^{22}}⊗\cdots ⊗\rho_{A^{2p_{2}}A^{3p_{3}}}\right)], \end{array}$$

where $M_{a_{11}|x_{11}}$ denotes the specific measurement operator of party A${}^{11}$ corresponding to the measurement choice $x_{11}$ with the outcome $a_{11}$, and other measurement operators have similar meanings.

In what follows, we examine quantum violations of the $\left(t_{3}-1\right)$-local inequalities (5) from pure states and mixed states, respectively.

**Non-($t_{3}$-1)-local correlations from pure states:** Firstly, let all $\left(t_{3}-1\right)$ sources produce any pure entangled states. Then, $\rho_{A^{11}A^{21}}$ can be written in the Schmidt basis as $\rho_{A^{11}A^{21}}=|\psi_{A^{11}A^{21}}\rangle \langle \psi_{A^{11}A^{21}}|$ with $|\psi_{A^{11}A^{21}}\rangle =b_{10}|00\rangle +b_{11}|11\rangle$ and $b_{10},b_{11}>0$, the normalized two-qubit pure state shared by the parties A${}^{11}$ and A${}^{21}$. Likewise, write $\rho_{A^{11}A^{2i}}=|\psi_{A^{11}A^{2i}}\rangle \langle \psi_{A^{11}A^{2i}}|$, $\forall i\in \{2,\cdots ,p_{2}\}$, $\rho_{A^{2j}A^{3k_{j}}}=|\psi_{A^{2j}A^{3k_{j}}}\rangle \langle \psi_{A^{2j}A^{3k_{j}}}|$, $\forall j\in \{1,2,\cdots ,p_{2}\}$, $k_{j}\in \left\{l_{2(j-1)}+1,\cdots ,l_{2j}\right\}$, where $|\psi_{A^{11}A^{2i}}\rangle =b_{i0}|00\rangle +b_{i1}|11\rangle$ and $|\psi_{A^{2j}A^{3k_{j}}}\rangle =c_{k_{j}0}|00\rangle +c_{k_{j}1}|11\rangle$ are also written in the Schmidt basis with the corresponding positive coefficients.

For party A${}^{11}$, take the measurement $A_{0}^{11}={⊗}_{k=1}^{p_{2}}\sigma_{z}^{k}$ and $A_{1}^{11}={⊗}_{k=1}^{p_{2}}\sigma_{x}^{k}$; for parties A${}^{2i}$ ($i=1,\cdots ,p_{2}$), the corresponding measurement choices are $A_{0}^{2i}={⊗}_{k=1}^{l_{2i}-l_{2(i-1)}+1}\sigma_{z}^{k}$ and $A_{1}^{2i}={⊗}_{k=1}^{l_{2i}-l_{2(i-1)}+1}\sigma_{x}^{k}$. Here, $\sigma_{z}^{k}=\sigma_{z}$ and $\sigma_{x}^{k}=\sigma_{x}$ for all *k* are Pauli matrices. Let the settings of all parties A${}^{3q}$ ($q=1,\cdots ,p_{3}$) correspond to any projective measurements in the Z-X plane of the Bloch sphere. Thus, each measurement can be characterized by an angle. Write the observables of A${}^{3q}$ by $A_{0}^{3q}=\left(\sin\alpha_{q},0,\cos\alpha_{q}\right)\cdot \vec{\sigma }$, $A_{1}^{3q}=\left(\sin\alpha_{q}^{\prime },0,\cos\alpha_{q}^{\prime }\right)\cdot \vec{\sigma }$, where $\vec{\sigma }=\left(\sigma_{x},\sigma_{y},\sigma_{z}\right)$ is the vector of Pauli matrices and $\alpha_{q},\alpha_{q}^{\prime }\in \left[0,2\pi \right]$ for all $q\in \{1,\cdots ,p_{3}\}$. Note that, if the above Schmidt bases differ from the computational basis, then it would be sufficient to add local unitary rotations to recover the case we discuss here. Then, we have

$$\begin{array}{cc} & \langle A_{0}^{11}A_{0}^{21}\cdots A_{0}^{2p_{2}}A_{0}^{31}\cdots A_{0}^{3p_{3}}\rangle \\ = & \mathrm{tr}\{\left[{⊗}_{k=1}^{p_{2}}\left(\sigma_{z}^{k}⊗\sigma_{z}^{k}\right)⊗\left(\sigma_{z}⊗A_{0}^{31}\right)⊗\cdots ⊗\left(\sigma_{z}⊗A_{0}^{3p_{3}}\right)\right]\\ & \cdot (\rho_{A^{11}A^{21}}⊗\cdots ⊗\rho_{A^{11}A^{2p_{2}}}⊗\rho_{A^{21}A^{31}}⊗\cdots ⊗\rho_{A^{2p_{2}}A^{3p_{3}}})\}\\ = & \prod_{i=1}^{p_{2}}\mathrm{tr}\left[\left(\sigma_{z}⊗\sigma_{z}\right)\rho_{A^{11}A^{2i}}\right]\mathrm{tr}\left[\left(\sigma_{z}⊗A_{0}^{31}\right)\rho_{A^{21}A^{31}}\right]\cdots \mathrm{tr}\left[\left(\sigma_{z}⊗A_{0}^{3p_{3}}\right)\rho_{A^{2p_{2}}A^{3p_{3}}}\right]\\ = & \cos\alpha_{1}\cos\alpha_{2}\cdots \cos\alpha_{p_{3}}. \end{array}$$

For any $x_{31},x_{32},\cdots ,x_{3p_{3}}\in \left\{0,1\right\}$, we can follow similar calculations as above for $\langle A_{0}^{11}A_{0}^{21}\cdots A_{0}^{2p_{2}}A_{x_{31}}^{31}\cdots A_{x_{3p_{3}}}^{3p_{3}}\rangle$ and $\langle A_{1}^{11}A_{1}^{21}\cdots A_{1}^{2p_{2}}A_{x_{31}}^{31}\cdots A_{x_{3p_{3}}}^{3p_{3}}\rangle$. Therefore,

$$\begin{array}{cc} I_{0,\cdots ,0,0}= & \frac{1}{2^{p_{3}}}\sum_{x_{31},\cdots ,x_{3p_{3}}}\langle A_{0}^{11}A_{0}^{21}\cdots A_{0}^{2p_{2}}A_{x_{31}}^{31}\cdots A_{x_{3p_{3}}}^{3p_{3}}\rangle \\ = & \frac{1}{2^{p_{3}}}\langle A_{0}^{11}A_{0}^{21}\cdots A_{0}^{2p_{2}}\left(A_{0}^{31}+A_{1}^{31}\right)\cdots \left(A_{0}^{3p_{3}}+A_{1}^{3p_{3}}\right)\rangle \\ = & \frac{1}{2^{p_{3}}}\left(\cos\alpha_{1}+\cos\alpha_{1}^{\prime }\right)\cdots \left(\cos\alpha_{p_{3}}+\cos\alpha_{p_{3}}^{\prime }\right) \end{array}$$

and

$$\begin{array}{cc} I_{1,\cdots ,1,1}= & \frac{1}{2^{p_{3}}}\sum_{x_{31},\cdots ,x_{3p_{3}}}{\left(-1\right)}^{x_{31}+\cdots +x_{3p_{3}}}\langle A_{1}^{11}A_{1}^{21}\cdots A_{1}^{2p_{2}}A_{x_{31}}^{31}\cdots A_{x_{3p_{3}}}^{3p_{3}}\rangle \\ = & \frac{1}{2^{p_{3}}}\langle A_{1}^{11}A_{1}^{21}\cdots A_{1}^{2p_{2}}\left(A_{0}^{31}-A_{1}^{31}\right)\cdots \left(A_{0}^{3p_{3}}-A_{1}^{3p_{3}}\right)\rangle \\ = & \frac{1}{2^{p_{3}}}\Delta \left(\sin\alpha_{1}-\sin\alpha_{1}^{\prime }\right)\cdots \left(\sin\alpha_{p_{3}}-\sin\alpha_{p_{3}}^{\prime }\right), \end{array}$$

where $\Delta =b^{\left(1\right)}\cdots b^{\left(p_{2}\right)}c^{\left(1\right)}\cdots c^{\left(p_{3}\right)}>0$, $b^{\left(i\right)}=2b_{i0}b_{i1}$, $i\in \{1,\cdots ,p_{2}\}$, $c^{\left(q\right)}=2c_{q0}c_{q1}$, $q\in \{1,\cdots ,p_{3}\}$. Consequently,

$$\begin{array}{cc} S_{(t_{3}-1)-\mathrm{local}}= & |I_{0,\cdots ,0,0}{|}^{\frac{1}{p_{3}}}+{\left|I_{1,\cdots ,1,1}\right|}^{\frac{1}{p_{3}}}\\ = & \frac{1}{2}{\left|\left(\cos\alpha_{1}+\cos\alpha_{1}^{\prime }\right)\cdots \left(\cos\alpha_{p_{3}}+\cos\alpha_{p_{3}}^{\prime }\right)\right|}^{\frac{1}{p_{3}}}\\ & +\frac{1}{2}{\left|\Delta \left(\sin\alpha_{1}-\sin\alpha_{1}^{\prime }\right)\cdots \left(\sin\alpha_{p_{3}}-\sin\alpha_{p_{3}}^{\prime }\right)\right|}^{\frac{1}{p_{3}}}. \end{array}$$

Write $S_{(t_{3}-1)-\mathrm{local}}=f\left(\alpha_{1},\alpha_{1}^{\prime },\cdots ,\alpha_{p_{3}},\alpha_{p_{3}}^{\prime }\right)$. To derive the maximum of differentiable function $f(\alpha_{1},\alpha_{1}^{\prime },\cdots ,\alpha_{p_{3}},\alpha_{p_{3}}^{\prime })$, we calculate all the partial derivatives $\frac{\partial f}{\partial \alpha_{i}}=0$, $\frac{\partial f}{\partial \alpha_{i}^{\prime }}=0$ for $i=1,2,\cdots ,p_{3}$. It follows that the extremal points of *f* must satisfy the conditions $\alpha_{i}=-\alpha_{i}^{\prime }$ and $|\tan\alpha_{i}|=\Delta^{1/p_{3}}$ ($i=1,2,\cdots ,p_{3}$). These force $|\cos\alpha_{i}|=|\cos\alpha_{i}^{\prime }|=\frac{1}{\sqrt{1+\Delta^{2/p_{3}}}}$ and $|\sin\alpha_{i}|=|\sin\alpha_{i}^{\prime }|=\frac{\Delta^{1/p_{3}}}{\sqrt{1+\Delta^{2/p_{3}}}}$, $i=1,2,\cdots ,p_{3}$. Therefore, the value of *f* at these extremal points is $|\cos\alpha_{1}|+\Delta^{1/p_{3}}\left|\sin\alpha_{1}\right|=\sqrt{1+\Delta^{2/p_{3}}}$. Comparing this value with the values of *f* at all boundary points, it is easily seen that the maximum of $S_{(t_{3}-1)-\mathrm{local}}$ is

(7) $$S_{(t_{3}-1)-\mathrm{local}}^{\max}=\sqrt{1+\Delta^{2/p_{3}}}>1.\;$$

Notice that $\mathrm{tr}\left[\left(\sigma_{z}⊗\sigma_{x}\right)\rho_{i}\right]=\mathrm{tr}\left[\left(\sigma_{x}⊗\sigma_{z}\right)\rho_{i}\right]$ = 0 hold for all $i=A^{11}A^{21},\cdots ,A^{2p_{2}}A^{3p_{3}}$. Thus, other possible nonzero terms for $|I_{i_{1},\cdots ,i_{t_{2}},0}{|}^{\frac{1}{p_{3}}}+{\left|I_{j_{1},\cdots ,j_{t_{2}},1}\right|}^{\frac{1}{p_{3}}}$ are $|I_{0,\cdots ,0,0}{|}^{\frac{1}{p_{3}}}+{\left|I_{0,\cdots ,0,1}\right|}^{\frac{1}{p_{3}}}$, $|I_{1,\cdots ,1,0}{|}^{\frac{1}{p_{3}}}+{\left|I_{1,\cdots ,1,1}\right|}^{\frac{1}{p_{3}}}$, and $|I_{1,\cdots ,1,0}{|}^{\frac{1}{p_{3}}}+{\left|I_{0,\cdots ,0,1}\right|}^{\frac{1}{p_{3}}}$. However, by similar discussions to the above, one can obtain that these three values are less than 1.

Hence, if all $\left(t_{3}-1\right)$ sources in the network of Figure 2 emit pure entangled states, they necessarily violate the $\left(t_{3}-1\right)$-local inequalities (5) and, thus, generate non-$\left(t_{3}-1\right)$-local correlations.

**Non-($t_{3}$-1)-local correlations from mixed states:** Now, we consider the case that all the sources in the network of Figure 2 produce any mixed states.

Assume that the state $\rho_{A^{11}A^{21}}$ shared by the parties A${}^{11}$ and A${}^{21}$ is a mixed state. Then, it has the following form:

$$\rho_{A^{11}A^{21}}=\frac{1}{4}\left(I⊗I+{\vec{r}}_{A^{11}}\cdot \vec{\sigma }⊗I+I⊗{\vec{r}}_{A^{21}}\cdot \vec{\sigma }+\sum_{i,j}t_{ij}^{A^{11}A^{21}}\sigma_{i}⊗\sigma_{j}\right),$$

where $\vec{\sigma }=\left(\sigma_{x},\sigma_{y},\sigma_{z}\right)$, ${\vec{r}}_{A^{11}}$ (${\vec{r}}_{A^{21}}$) represents the Bloch vector of the reduced state of subsystem A${}^{11}$ (A${}^{21}$), and $T^{A^{11}A^{21}}=\left(t_{ij}^{A^{11}A^{21}}\right)$ with i,j∈{x,y,z} is the correlation matrix. By the polar decomposition, the correlation matrix $T^{A^{11}A^{21}}$ can be written as $T^{A^{11}A^{21}}=U^{A^{11}A^{21}}R^{A^{11}A^{21}}$, where $U^{A^{11}A^{21}}$ is a unitary matrix and $R^{A^{11}A^{21}}=\sqrt{{\left(T^{A^{11}A^{21}}\right)}^{†}T^{A^{11}A^{21}}}\geq 0$. Denote by $\sqrt{\tau_{1}^{A^{11}A^{21}}}\geq \sqrt{\tau_{2}^{A^{11}A^{21}}}\geq \sqrt{\tau_{3}^{A^{11}A^{21}}}\geq 0$ the three non-negative eigenvalues of $R^{A^{11}A^{21}}$.

For the other mixed states $\rho_{A^{11}A^{22}},\cdots ,\rho_{A^{11}A^{2p_{2}}},\rho_{A^{21}A^{31}},\cdots ,\rho_{A^{2p_{2}}A^{3p_{3}}}$, shared by the corresponding parties, they also have similar expressions to that of $\rho_{A^{11}A^{21}}$, and the corresponding matrices and the eigenvalues are, respectively, represented as

$$R^{A^{11}A^{2i}}=\sqrt{{\left(T^{A^{11}A^{2i}}\right)}^{†}T^{A^{11}A^{2i}}},\;\;\sqrt{\tau_{1}^{A^{11}A^{2i}}}\geq \sqrt{\tau_{2}^{A^{11}A^{2i}}}\geq \sqrt{\tau_{3}^{A^{11}A^{2i}}}\geq 0,$$

$$R^{A^{2j}A^{3k_{j}}}=\sqrt{{\left(T^{A^{2j}A^{3k_{j}}}\right)}^{†}T^{A^{2j}A^{3k_{j}}}},\;\;\sqrt{\tau_{1}^{A^{2j}A^{3k_{j}}}}\geq \sqrt{\tau_{2}^{A^{2j}A^{3k_{j}}}}\geq \sqrt{\tau_{3}^{A^{2j}A^{3k_{j}}}}\geq 0,$$

where $i\in \{1,2,\cdots ,p_{2}\}$, $k_{j}\in \left\{l_{2(j-1)}+1,\cdots ,l_{2j}\right\}$, and $j\in \{1,2,\cdots ,p_{2}\}$.

Suppose that party A${}^{11}$ performs measurements $A_{0}^{11}={⊗}_{k=1}^{p_{2}}\sigma_{z}^{k}$, $A_{1}^{11}={⊗}_{k=1}^{p_{2}}\sigma_{x}^{k}$. We consider the Z and X Bloch directions (on the first subsystem of party A${}^{11}$, connected to the first subsystem of party A${}^{21}$) given by the eigenvectors of the matrix $R^{A^{11}A^{21}}$ corresponding to the two largest eigenvalues $\sqrt{\tau_{1}^{A^{11}A^{21}}}$ and $\sqrt{\tau_{2}^{A^{11}A^{21}}}$, respectively [19]. Similarly, we use $R^{A^{11}A^{2i}}$ for aligning the *i*th subsystem of A${}^{11}$, connected to the first subsystem of A${}^{2i}$, $i=2,\cdots ,p_{2}$. Note that the Z and X axes used by the parties A${}^{11}$ and A${}^{2i}$ ($i=1,\cdots ,p_{2}$) may be different from each other. In this case, party A${}^{11}$ can perform different unitary transformations to the $p_{2}$ qubits she/he shares with A${}^{21}$, ⋯, A${}^{2p_{2}}$ before performing the measurements. Likewise, we may assume the party A${}^{2i}$ ($i=1,\cdots ,p_{2}$) has measurement choices $A_{0}^{2i}={⊗}_{k=1}^{l_{2i}-l_{2(i-1)}+1}\sigma_{z}^{k}$ and $A_{1}^{2i}={⊗}_{k=1}^{l_{2i}-l_{2(i-1)}+1}\sigma_{x}^{k}$. For party A${}^{3q}$ ($q=1,\cdots ,p_{3}$), he/she performs projective measurements on the Z and X Bloch directions, which are composed of the two eigenvectors with largest eigenvalues of the connected matrix $R^{A^{2j_{q}}A^{3q}}$ ($1\leq j_{q}\leq p_{2}$). That is, $A_{0}^{3q}=\vec{c_{q}}\cdot \vec{\sigma }$ and $A_{1}^{3q}={\vec{c_{q}}}^{\prime }\cdot \vec{\sigma }$, where $\vec{c_{q}}=\left(\sin\beta_{q},0,\cos\beta_{q}\right)$, ${\vec{c_{q}}}^{\prime }=\left(\sin\beta_{q}^{\prime },0,\cos\beta_{q}^{\prime }\right)$, $\beta_{q},\beta_{q}^{\prime }\in \left[0,2\pi \right]$.

Now, we have

$$\begin{array}{cc} I_{0,\cdots ,0,0}= & \frac{1}{2^{p_{3}}}\sum_{x_{31},\cdots ,x_{3p_{3}}}\langle A_{0}^{11}A_{0}^{21}\cdots A_{0}^{2p_{2}}A_{x_{31}}^{31}\cdots A_{x_{3p_{3}}}^{3p_{3}}\rangle \\ = & \frac{1}{2^{p_{3}}}\langle A_{0}^{11}A_{0}^{21}\cdots A_{0}^{2p_{2}}\left(A_{0}^{31}+A_{1}^{31}\right)\cdots \left(A_{0}^{3p_{3}}+A_{1}^{3p_{3}}\right)\rangle \\ = & \frac{1}{2^{p_{3}}}\langle \left({⊗}_{k=1}^{p_{2}}\sigma_{z}^{k}\right)⊗\left({⊗}_{k=1}^{l_{21}+1}\sigma_{z}^{k}\right)⊗\cdots ⊗\left({⊗}_{k=1}^{l_{2p_{2}}-l_{2(p_{2}-1)}+1}\sigma_{z}^{k}\right)⊗\left[\left(\vec{c_{1}}+{\vec{c_{1}}}^{\prime }\right)\cdot \vec{\sigma }\right]⊗\cdots \\ & ⊗[\left(\vec{c_{p_{3}}}+{\vec{c_{p_{3}}}}^{\prime }\right)\cdot \vec{\sigma }]\rangle \\ = & \frac{1}{2^{p_{3}}}\mathrm{tr}\{\left[\left({⊗}_{k=1}^{p_{2}}\left(\sigma_{z}^{k}⊗\sigma_{z}^{k}\right)\right)⊗\left(\sigma_{z}⊗\left(\vec{c_{1}}+{\vec{c_{1}}}^{\prime }\right)\cdot \vec{\sigma }\right)⊗\cdots ⊗\left(\sigma_{z}⊗\left(\vec{c_{p_{3}}}+{\vec{c_{p_{3}}}}^{\prime }\right)\cdot \vec{\sigma }\right)\right]\\ & \cdot (\rho_{A^{11}A^{21}}⊗\cdots ⊗\rho_{A^{11}A^{2p_{2}}}⊗\rho_{A^{21}A^{31}}⊗\cdots ⊗\rho_{A^{2p_{2}}A^{3p_{3}}})\}\\ = & \frac{1}{2^{p_{3}}}\mathrm{tr}\left[\left(\sigma_{z}⊗\sigma_{z}\right)\rho_{A^{11}A^{21}}\right]\cdots \mathrm{tr}\left[\left(\sigma_{z}⊗\sigma_{z}\right)\rho_{A^{11}A^{2p_{2}}}\right]\mathrm{tr}\left[\left(\sigma_{z}⊗\left(\vec{c_{1}}+{\vec{c_{1}}}^{\prime }\right)\cdot \vec{\sigma }\right)\rho_{A^{21}A^{31}}\right]\\ & \cdots \mathrm{tr}[\left(\sigma_{z}⊗\left(\vec{c_{p_{3}}}+{\vec{c_{p_{3}}}}^{\prime }\right)\cdot \vec{\sigma }\right)\rho_{A^{2p_{2}}A^{3p_{3}}}]\\ = & \frac{1}{2^{p_{3}}}\sqrt{\tau_{1}^{A^{11}A^{21}}\cdots \tau_{1}^{A^{11}A^{2p_{2}}}\tau_{1}^{A^{21}A^{31}}\cdots \tau_{1}^{A^{2p_{2}}A^{3p_{3}}}}\prod_{j=1}^{p_{3}}\left(\cos\beta_{j}+\cos\beta_{j}^{\prime }\right) \end{array}$$

and

$$\begin{array}{cc} I_{1,\cdots ,1,1}= & \frac{1}{2^{p_{3}}}\sum_{x_{31},\cdots ,x_{3p_{3}}}{\left(-1\right)}^{x_{31}+\cdots +x_{3p_{3}}}\langle A_{1}^{11}A_{1}^{21}\cdots A_{1}^{2p_{2}}A_{x_{31}}^{31}\cdots A_{x_{3p_{3}}}^{3p_{3}}\rangle \\ = & \frac{1}{2^{p_{3}}}\langle A_{1}^{11}A_{1}^{21}\cdots A_{1}^{2p_{2}}\left(A_{0}^{31}-A_{1}^{31}\right)\cdots \left(A_{0}^{3p_{3}}-A_{1}^{3p_{3}}\right)\rangle \\ = & \frac{1}{2^{p_{3}}}\sqrt{\tau_{2}^{A^{11}A^{21}}\cdots \tau_{2}^{A^{11}A^{2p_{2}}}\tau_{2}^{A^{21}A^{31}}\cdots \tau_{2}^{A^{2p_{2}}A^{3p_{3}}}}\prod_{j=1}^{p_{3}}\left(\sin\beta_{j}-\sin\beta_{j}^{\prime }\right), \end{array}$$

and so

$$\begin{array}{cc} S_{(t_{3}-1)-\mathrm{local}}= & |I_{0,\cdots ,0,0}{|}^{\frac{1}{p_{3}}}+{\left|I_{1,\cdots ,1,1}\right|}^{\frac{1}{p_{3}}}\\ = & \frac{1}{2}{\left(\tau_{1}^{A^{11}A^{21}}\cdots \tau_{1}^{A^{11}A^{2p_{2}}}\tau_{1}^{A^{21}A^{31}}\cdots \tau_{1}^{A^{2p_{2}}A^{3p_{3}}}\right)}^{\frac{1}{2p_{3}}}{\left|\prod_{j=1}^{p_{3}}\left(\cos\beta_{j}+\cos\beta_{j}^{\prime }\right)\right|}^{\frac{1}{p_{3}}}\\ & +\frac{1}{2}{\left(\tau_{2}^{A^{11}A^{21}}\cdots \tau_{2}^{A^{11}A^{2p_{2}}}\tau_{2}^{A^{21}A^{31}}\cdots \tau_{2}^{A^{2p_{2}}A^{3p_{3}}}\right)}^{\frac{1}{2p_{3}}}|\prod_{j=1}^{p_{3}}{\left(\sin\beta_{j}-\sin\beta_{j}^{\prime }\right|}^{\frac{1}{p_{3}}}. \end{array}$$

A calculation gives the maximum

(8) $$S_{(t_{3}-1)-\mathrm{local}}^{\max}=\sqrt{\sum_{i=1}^{2}{\left(\tau_{i}^{A^{11}A^{21}}\cdots \tau_{i}^{A^{11}A^{2p_{2}}}\tau_{i}^{A^{21}A^{31}}\cdots \tau_{i}^{A^{2p_{2}}A^{3p_{3}}}\right)}^{\frac{1}{p_{3}}}}.\;$$

The detailed proof for Equation (8) is in Appendix A.

It is easily verified that the above Equation (8) reduces to Equation (7) for the case of pure states discussed in the above. In fact, for pure states, we have $\tau_{1}^{A^{11}A^{21}}=\cdots =\tau_{1}^{A^{11}A^{2p_{2}}}=\tau_{1}^{A^{21}A^{31}}=\cdots =\tau_{1}^{A^{2p_{2}}A^{3p_{3}}}=1$ and $\tau_{2}^{A^{11}A^{21}}={\left(b^{\left(1\right)}\right)}^{2}$, ⋯, $\tau_{2}^{A^{11}A^{2p_{2}}}={\left(b^{\left(p_{2}\right)}\right)}^{2}$, $\tau_{2}^{A^{21}A^{31}}={\left(c^{\left(1\right)}\right)}^{2}$, ⋯, $\tau_{2}^{A^{2p_{2}}A^{3p_{3}}}={\left(c^{\left(p_{3}\right)}\right)}^{2}$, which implies that Equation (8) can be reduced to Equation (7).

From Equation (8), $S_{(t_{3}-1)-\mathrm{local}}^{\max}>1$ implies that these states violate the $\left(t_{3}-1\right)$-locality inequalities (5). Since all these eigenvalues $\tau_{i}^{A^{11}A^{21}},\cdots ,\tau_{i}^{A^{2p_{2}}A^{3p_{3}}}$ (i=1,2) belong to [0,1] ([23] Lemma 3), by [21] Lemma 1, Equation (8) implies

$$\begin{array}{cc} S_{(t_{3}-1)-\mathrm{local}}^{\max}\leq & \sqrt{\sum_{i=1}^{2}{\left(\tau_{i}^{A^{11}A^{21}}\cdots \tau_{i}^{A^{11}A^{2p_{2}}}\tau_{i}^{A^{21}A^{31}}\cdots \tau_{i}^{A^{2p_{2}}A^{3p_{3}}}\right)}^{\frac{1}{p_{2}+p_{3}}}}\\ \leq & (\sqrt{\tau_{1}^{A^{11}A^{21}}+\tau_{2}^{A^{11}A^{21}}}\cdots \sqrt{\tau_{1}^{A^{11}A^{2p_{2}}}+\tau_{2}^{A^{11}A^{2p_{2}}}}\sqrt{\tau_{1}^{A^{21}A^{31}}+\tau_{2}^{A^{21}A^{31}}}\\ & \cdots \sqrt{\tau_{1}^{A^{2p_{2}}A^{3p_{3}}}+\tau_{2}^{A^{2p_{2}}A^{3p_{3}}}}{\;)}^{\frac{1}{p_{2}+p_{3}}}\\ ≐ & {\left(S_{A^{11}A^{21}}^{\max}\cdots S_{A^{11}A^{2p_{2}}}^{\max}S_{A^{21}A^{31}}^{\max}\cdots S_{A^{2p_{2}}A^{3p_{3}}}^{\max}\right)}^{\frac{1}{p_{2}+p_{3}}}, \end{array}$$

where the expressions $S_{XY}^{\max}=\sqrt{\tau_{1}^{XY}+\tau_{2}^{XY}}$ represent the maximal CHSH value for the corresponding state $\rho_{XY}$ by the Horodecki criterion in [43]. From the above inequality, we know that once the states altogether violate the $\left(t_{3}-1\right)$-locality inequalities, at least one of these states necessarily violates the CHSH inequality. However, for each state violating the CHSH inequality, this does not imply that it necessarily violates the $\left(t_{3}-1\right)$-locality inequalities. We illustrate this case by the specific network of Figure 3. For example, let nine sources in Figure 3 all produce the following state:

$$\rho =\frac{3}{4}|\psi^{-}\rangle \langle \psi^{-}|+\frac{1}{20}(|\psi^{+}\rangle \langle \psi^{+}|+I)=\left(\begin{array}{cccc} 0.05 & 0 & 0 & 0\\ 0 & 0.45 & -0.35 & 0\\ 0 & -0.35 & 0.45 & 0\\ 0 & 0 & 0 & 0.05 \end{array}\right),$$

where $|\psi^{\pm }\rangle =(|01\rangle \pm |10\rangle )/\sqrt{2}$. It is easily obtained that $\tau_{1}=0.64$ and $\tau_{2}=0.49$. For this single state, the maximal CHSH value is $S^{\max}≃1.06>1.$ However, through distributing nine copies of this state in the network of Figure 3, the maximal value of the corresponding nine-local inequality is $S_{9-\mathrm{local}}^{\max}≃0.98<1.$

**Remark** **1.** *To achieve the maximal quantum violation of the $\left(t_{3}-1\right)$-local correlation inequalities (5), all possible quantum measurements should be considered. However, this is almost impossible because the calculation is very difficult and complicated. Therefore, for the network of Figure 2, we take separable measurements for parties A${}^{1j}$ and A${}^{2j}$ and any measurements for parties A${}^{3j}$. In this quantum strategy, the maximal violation $S_{(t_{3}-1)-\mathrm{local}}^{\max}$ is obtained, which gives a sufficient condition that $S_{(t_{3}-1)-\mathrm{local}}^{\max}>1$ ensures that the state ρ violates the inequality (5) and, thus, is non-$\left(t_{3}-1\right)$-local. Of course, there are other strategies of the measurement choices that are computable, and some of them may be better than our strategy, though we have not discovered them yet. This is an interesting problem that is worth being explored later.*

#### 2.1.3. Resistance to White Noise

Now, suppose that each source $S_{i}$ ($i=1,\cdots ,t_{3}-1$) in Figure 2 produces Bell state $|\phi^{+}\rangle =(|00\rangle +|11\rangle )/\sqrt{2}$ with white noise of probability $1-v_{i}$. Then, the state it actually produces is the Werner state of the form

$$\rho_{i}\left(v_{i}\right)=v_{i}|\phi^{+}\rangle \langle \phi^{+}|+\left(1-v_{i}\right)\frac{I}{4}.$$

Let the input of party A${}^{11}$ be $\left\{A_{0}^{11}={⊗}_{k=1}^{p_{2}}\sigma_{z}^{k},\;A_{1}^{11}={⊗}_{k=1}^{p_{2}}\sigma_{x}^{k}\right\}$. For party A${}^{2j}$ ($j=1,\cdots ,p_{2}$), the measurement choices are $\left\{A_{0}^{2j}={⊗}_{k=1}^{l_{2j}-l_{2(j-1)}+1}\sigma_{z}^{k},\;A_{1}^{2j}={⊗}_{k=1}^{l_{2j}-l_{2(j-1)}+1}\sigma_{x}^{k}\right\}$. Suppose the inputs of each party A${}^{3q}$ ($q=1,2,\cdots ,p_{3}$) are measurements $\left\{A_{0}^{3q}=\left(\sigma_{z}+\sigma_{x}\right)/\sqrt{2},\;A_{1}^{3q}=\left(\sigma_{z}-\sigma_{x}\right)/\sqrt{2}\right\}$. Denoting by $V=\prod_{i=1}^{t_{3}-1}v_{i}$ as the overall visibility, we obtain $I_{0,\cdots ,0,0}={\left(\frac{1}{\sqrt{2}}\right)}^{p_{3}}V$, $I_{1,\cdots ,1,1}={\left(\frac{1}{\sqrt{2}}\right)}^{p_{3}}V$, and so,

$$|I_{0,\cdots ,0,0}{|}^{\frac{1}{p_{3}}}+{\left|I_{1,\cdots ,1,1}\right|}^{\frac{1}{p_{3}}}=\sqrt{2}V^{1/p_{3}}.$$

That is to say, $V>{\left(\frac{1}{\sqrt{2}}\right)}^{p_{3}}$ implies non-$\left(t_{3}-1\right)$-local correlations. Assuming that all the $\left(t_{3}-1\right)$ sources emit states with the same noise parameter $v_{i}=v$, we thus see that a single source necessarily satisfies $v>{\left(\frac{1}{\sqrt{2}}\right)}^{\frac{p_{3}}{t_{3}-1}}$, which is a little greater than $v^{\prime }>\frac{1}{\sqrt{2}}$ for the Werner state to violate the CHSH inequality.

### 2.2. $\left(t_{n}-1\right)$-Local Network Scenario

In this subsection, we consider the nonlocality of the general tree-shaped network of Figure 1. With similar arguments, if each party A${}^{i}$$\left(i=11,21,\cdots ,np_{n}\right)$ has binary input $x_{i}\left(\in \left\{0,1\right\}\right)$ with binary output $a_{i}\left(\in \left\{0,1\right\}\right)$, then we obtain the following results for any n≥2.

**Theorem** **3.** *All $\left(t_{n}-1\right)$-local correlations generated by the tree-shaped network of Figure 1 necessarily satisfy the following inequalities:*

(9) $$|I_{i_{1},\cdots ,i_{t_{n-1}},0}{|}^{\frac{1}{p_{n}}}+{\left|I_{j_{1},\cdots ,j_{t_{n-1}},1}\right|}^{\frac{1}{p_{n}}}\leq 1,\;\;\forall i_{1},\cdots ,i_{t_{n-1}},j_{1},\cdots ,j_{t_{n-1}}\in \left\{0,1\right\};\;$$

*and the corresponding local correlations satisfy the following inequalities:*

(10) $$|I_{i_{1},\cdots ,i_{t_{n-1}},0}\left|+\right|I_{j_{1},\cdots ,j_{t_{n-1}},1}|\leq 1,\;\;\forall i_{1},\cdots ,i_{t_{n-1}},j_{1},\cdots ,j_{t_{n-1}}\in \left\{0,1\right\}.\;$$

*where for k∈{0,1},*

$$\begin{array}{cc} & I_{i_{1}\left(j_{1}\right),\cdots ,i_{t_{n-1}}\left(j_{t_{n-1}}\right),k}\\ = & \frac{1}{2^{p_{n}}}\sum_{x_{n1},\cdots ,x_{np_{n}}}{\left(-1\right)}^{k\cdot \left(x_{n1}+\cdots +x_{np_{n}}\right)}\langle A_{i_{1}\left(j_{1}\right)}^{11}\cdots A_{i_{t_{n-1}}\left(j_{t_{n-1}}\right)}^{\left(n-1\right)p_{n-1}}A_{x_{n1}}^{n1}\cdots A_{x_{np_{n}}}^{np_{n}}\rangle , \end{array}$$

$$\begin{array}{cc} & \langle A_{x_{11}}^{11}\cdots A_{x_{\left(n-1\right)p_{n-1}}}^{\left(n-1\right)p_{n-1}}A_{x_{n1}}^{n1}\cdots A_{x_{np_{n}}}^{np_{n}}\rangle \\ = & \sum_{a_{11},\cdots ,a_{np_{n}}}{\left(-1\right)}^{m}P\left(a_{11},\cdots ,a_{\left(n-1\right)p_{n-1}},a_{n1},\cdots ,a_{np_{n}}|x_{11},\cdots ,x_{\left(n-1\right)p_{n-1}},x_{n1},\cdots ,x_{np_{n}}\right), \end{array}$$

*$m=a_{11}+\cdots +a_{np_{n}}$, and $A_{x_{i}}^{i}$ denotes the observable for binary inputs $x_{i}$ of party A${}^{i}$, $i=11,21,22,\cdots ,np_{n}$.*

Note that the subscript $t_{n-1}$ in Ineqs. (9) and (10) represents the total number of parties $A^{11}$, $A^{21}$, ⋯, $A^{2p_{2}}$, ⋯, $A^{(n-1)1}$, ⋯, $A^{\left(n-1\right)p_{n-1}}$. In particular, if n=3, then Inequalities (9) and (10) reduce to Inequalities (5) and (6), respectively. By Theorem 3, violating Inequalities (9) for at least one possible $(i_{1},\cdots ,i_{t_{n-1}}$, $j_{1},\cdots ,j_{t_{n-1}})$ implies the non-$\left(t_{n}-1\right)$-local nature of the general tree-shaped networks in Figure 1. The proof of Theorem 3 is provided in Appendix A.

Next, assume that all sources in Figure 1 produce pure entangled states $|\psi_{i}\rangle \langle \psi_{i}|$, $|\psi_{i}\rangle =b_{i0}|00\rangle +b_{i1}|11\rangle$, written in the Schmidt basis, with positive coefficients $b_{i0}$ and $b_{i1}$, $i=1,2,\cdots ,t_{n}-1$. Let the measurements of A${}^{11}$ be $\left\{A_{0}^{11}={⊗}_{k=1}^{p_{2}}\sigma_{z}^{k},A_{1}^{11}={⊗}_{k=1}^{p_{2}}\sigma_{x}^{k}\right\}$. For parties A${}^{ij}$ with i=2,3,⋯,n−1 and $j=1,2,\cdots ,p_{i}$, they have the same measurement choices $\left\{A_{0}^{ij}={⊗}_{k=1}^{l_{ij}-l_{i(j-1)}+1}\sigma_{z}^{k},A_{1}^{ij}={⊗}_{k=1}^{l_{ij}-l_{i(j-1)}+1}\sigma_{x}^{k}\right\}$. Here, $\sigma_{z}^{k}=\sigma_{z}$ and $\sigma_{x}^{k}=\sigma_{z}$ for any *k*. For the parties A${}^{nq}$ ($q=1,2,\cdots ,p_{n}$), they perform projective measurements denoted by $\left\{A_{0}^{nq}=\left(\sin\alpha_{q},0,\cos\alpha_{q}\right)\cdot \vec{\sigma },A_{1}^{nq}=\left(\sin\alpha_{q}^{\prime },0,\cos\alpha_{q}^{\prime }\right)\cdot \vec{\sigma }\right\}$, where $\alpha_{q},\alpha_{q}^{\prime }\in \left[0,2\pi \right]$. With similar arguments to that of Equation (7), one obtains $I_{0,\cdots ,0,0}=\frac{1}{2^{p_{n}}}\left(\cos\alpha_{1}+\cos\alpha_{1}^{\prime }\right)\cdots \left(\cos\alpha_{p_{n}}+\cos\alpha_{p_{n}}^{\prime }\right)$ and $I_{1,\cdots ,1,1}=\frac{1}{2^{p_{n}}}\Delta \left(\sin\alpha_{1}-\sin\alpha_{1}^{\prime }\right)\cdots \left(\sin\alpha_{p_{n}}-\sin\alpha_{p_{n}}^{\prime }\right)$, where

$$\Delta =b^{\left(1\right)}b^{\left(2\right)}\cdots b^{\left(t_{n}-1\right)}>0,$$

$b^{\left(i\right)}=2b_{i0}b_{i1}$, $i=1,2,\cdots ,t_{n}-1$. Therefore, the maximum of $S_{(t_{n}-1)-\mathrm{local}}=|I_{0,\cdots ,0,0}{|}^{\frac{1}{p_{n}}}+{\left|I_{1,\cdots ,1,1}\right|}^{\frac{1}{p_{n}}}$ is

(11) $$S_{(t_{n}-1)-\mathrm{local}}^{\max}=\sqrt{1+\Delta^{2/p_{n}}}>1.\;$$

That is to say, all pure entangled states distributed in the network of Figure 1 indicate the non-$\left(t_{n}-1\right)$-local correlations.

Finally, we consider that all sources in Figure 1 produce any mixed states $\rho_{i}$, $i=1,2,\cdots ,t_{n}-1$. Let $T^{i}$ be the correlation matrix of $\rho_{i}$ and $\tau_{1}^{\left(i\right)}$, $\tau_{2}^{\left(i\right)}$ the two larger non-negative eigenvalues of ${\left(T^{i}\right)}^{†}T^{i}$, $i=1,2,\cdots ,t_{n}-1$. Let party A${}^{11}$ perform measurements $\left\{A_{0}^{11}={⊗}_{k=1}^{p_{2}}\sigma_{z}^{k},A_{1}^{11}={⊗}_{k=1}^{p_{2}}\sigma_{x}^{k}\right\}$; party A${}^{ij}$ perform measurements $\left\{A_{0}^{ij}={⊗}_{k=1}^{l_{ij}-l_{i(j-1)}+1}\sigma_{z}^{k},A_{1}^{ij}={⊗}_{k=1}^{l_{ij}-l_{i(j-1)}+1}\sigma_{x}^{k}\right\}$ (i=2,3,⋯,n−1, $j=1,2,\cdots ,p_{i}$); and party A${}^{nq}$ have measurement choices $\left\{A_{0}^{nq}=\left(\sin\beta_{q},0,\cos\beta_{q}\right)\cdot \vec{\sigma },A_{1}^{nq}=\left(\sin\beta_{q}^{\prime },0,\cos\beta_{q}^{\prime }\right)\cdot \vec{\sigma }\right\}$ with $\beta_{q},\beta_{q}^{\prime }\in \left[0,2\pi \right]$ ($q=1,2,\cdots ,p_{n}$). By calculations, we obtain

$$I_{0,\cdots ,0,0}=\frac{1}{2^{p_{n}}}\sqrt{\tau_{1}^{\left(1\right)}\tau_{1}^{\left(2\right)}\tau_{1}^{\left(3\right)}\cdots \tau_{1}^{\left(t_{n}-1\right)}}\prod_{i=1}^{p_{n}}\left(\cos\beta_{i}+\cos\beta_{i}^{\prime }\right)$$

and

$$I_{1,\cdots ,1,1}=\frac{1}{2^{p_{n}}}\sqrt{\tau_{2}^{\left(1\right)}\tau_{2}^{\left(2\right)}\tau_{2}^{\left(3\right)}\cdots \tau_{2}^{\left(t_{n}-1\right)}}\prod_{i=1}^{p_{n}}\left(\sin\beta_{i}-\sin\beta_{i}^{\prime }\right).$$

Following the analogous proof process of Equation (8), we have that the maximal value of $S_{(t_{n}-1)-\mathrm{local}}=|I_{0,\cdots ,0,0}{|}^{\frac{1}{p_{n}}}+{\left|I_{1,\cdots ,1,1}\right|}^{\frac{1}{p_{n}}}$ is

(12) $$S_{(t_{n}-1)-\mathrm{local}}^{\max}=\sqrt{{\left(\tau_{1}^{\left(1\right)}\tau_{1}^{\left(2\right)}\tau_{1}^{\left(3\right)}\cdots \tau_{1}^{\left(t_{n}-1\right)}\right)}^{1/(p_{n})}+{\left(\tau_{2}^{\left(1\right)}\tau_{2}^{\left(2\right)}\tau_{2}^{\left(3\right)}\cdots \tau_{2}^{\left(t_{n}-1\right)}\right)}^{1/(p_{n})}}.\;$$

When n=3, Equations (11) and (12) reduce to Equations (7) and (8). By Equation (12), $S_{(t_{n}-1)-\mathrm{local}}^{\max}>1$ implies the non-$\left(t_{n}-1\right)$-local correlations.

Besides, if all sources in Figure 1, respectively, distribute Werner states with visibilities $v_{1},v_{2},\cdots ,v_{t_{n}-1}$, then we take the inputs of A${}^{11}$$\left\{A_{0}^{11}={⊗}_{k=1}^{p_{2}}\sigma_{z}^{k},A_{1}^{11}={⊗}_{k=1}^{p_{2}}\sigma_{x}^{k}\right\}$; the inputs of A${}^{ij}$ $\left\{A_{0}^{ij}={⊗}_{k=1}^{l_{ij}-l_{i(j-1)}+1}\sigma_{z}^{k},A_{1}^{ij}={⊗}_{k=1}^{l_{ij}-l_{i(j-1)}+1}\sigma_{x}^{k}\right\}$ (i=2,3,⋯,n−1, $j=1,2,\cdots ,p_{i}$); and the inputs of A${}^{nq}$ $\left\{A_{0}^{nq}=\left(\sigma_{z}+\sigma_{x}\right)/\sqrt{2},A_{1}^{nq}=\left(\sigma_{z}-\sigma_{x}\right)/\sqrt{2}\right\}$. Let the overall visibility be $V=\prod_{i=1}^{t_{n}-1}v_{i}$. A calculation gives $I_{0,\cdots ,0,0}={\left(\frac{1}{\sqrt{2}}\right)}^{p_{n}}V$ and $I_{1,\cdots ,1,1}={\left(\frac{1}{\sqrt{2}}\right)}^{p_{n}}V$, and so,

$$|I_{0,\cdots ,0,0}{|}^{\frac{1}{p_{n}}}+{\left|I_{1,\cdots ,1,1}\right|}^{\frac{1}{p_{n}}}=\sqrt{2}V^{1/p_{n}}.$$

Hence, if $V>{\left(\frac{1}{\sqrt{2}}\right)}^{p_{n}}$, then the inequalities (9) will be violated, demonstrating non-$\left(t_{n}-1\right)$-local correlations in Figure 1.

### 2.3. Comparing Any Forked Tree-Shaped Network with Other Networks

In this subsection, we discuss the relationships of multi-local inequalities between any forked tree-shaped network of Figure 1 and a bilocal network, chain-shaped network, star-shaped network, and two-forked tree-shaped network.

In fact, when *n* = 2 and $p_{2}$ = 2, the network of Figure 1 reduces to a bilocal network and the ($t_{n}$-1)-local Ineq. (5) reduces to the bilocal Ineq. (20) in [18]. When $p_{2}=\cdots =p_{n}=2$, the network of Figure 1 reduces to the chain-shaped network and Ineq. (5) reduces to the (2n−2)-local Ineq. (16) in [20]. When *n* = 2, the network of Figure 1 reduces to a star-shaped network and Ineq. (5) reduces to the $p_{2}$-local Ineq. (7) in [21]. Moreover, if $l_{kj}-l_{k(j-1)}$ = 2 holds for any (k,j), then the network of Figure 1 reduces to a two-forked tree-shaped network and Ineq. (5) reduces to the ($2^{n}-2$)-local Ineq. (16) in [42]. See Table 1. Therefore, from this point of view, any forked tree-shaped network can be seen as a generalization of these networks.

## 3. Discussions

In this work, we discussed the nonlocality of a kind of important quantum network: any forked tree-shaped network, in which each node, respectively, shares an arbitrary number of bipartite sources with other nodes in the next “layer”. This network contains $\left(t_{n}-1\right)$ independent bipartite sources and $t_{n}$ noninteracting parties (n≥2). The “two-forked” tree-shaped networks discussed in [42] are special tree-shaped networks. In addition, if n=2, the networks are in fact the $p_{2}$-local star networks introduced in [21]. If $p_{2}=\cdots =p_{n}=2$, the networks are reduced to the chain networks introduced in [20]. We gave a detailed discussion for the case of n=3, i.e., a tree-shaped network scenario with $t_{3}$ particles and $\left(t_{3}-1\right)$ independent sources. Concretely, we gave the inequalities satisfied by all $\left(t_{3}-1\right)$-local correlations, proved that all pure entangled states violate these $\left(t_{3}-1\right)$-local inequalities, obtained a necessary condition for mixed states to violate these inequalities, and explored the relation between the $\left(t_{3}-1\right)$-locality correlation and locality correlation in this quantum network. Finally, we generalized these results to the general $t_{n}$-partite tree-shaped networks. Note that the tree-shaped networks examined here just involve bipartite quantum states. The nonlocality of tree-shaped networks with multipartite states deserves further research.

## Figures and Tables

**Table 1:** Comparison of multi-local inequalities between any forked tree-shaped network and other networks.

Networks	Multi-Local Inequalities	Relations
any forked tree-shaped	$|I_{i_{1},\cdots ,i_{t_{n-1}},0}{|}^{\frac{1}{p_{n}}}+{\left|I_{j_{1},\cdots ,j_{t_{n-1}},1}\right|}^{\frac{1}{p_{n}}}\leq 1$	
bilocal	$|I_{i_{1},0}{|}^{\frac{1}{2}}+{\left|I_{j_{1},1}\right|}^{\frac{1}{2}}\leq 1$	*n* = 2, $p_{2}$ = 2
chain-shaped	$|I_{i_{1},\cdots ,i_{2n-3},0}{|}^{\frac{1}{2}}+{\left|I_{j_{1},\cdots ,j_{2n-3},1}\right|}^{\frac{1}{2}}\leq 1$	$p_{2}=\cdots =p_{n}=2$
star-shaped	$|I_{i_{1},0}{|}^{\frac{1}{p_{2}}}+{\left|I_{j_{1},1}\right|}^{\frac{1}{p_{2}}}\leq 1$	*n* = 2
two-forked tree-shaped	$|I_{i_{1},\cdots ,i_{2^{n-1}-1},0}{|}^{\frac{1}{2^{n-1}}}+{\left|I_{j_{1},\cdots ,j_{2^{n-1}-1},1}\right|}^{\frac{1}{2^{n-1}}}\leq 1$	$l_{kj}-l_{k(j-1)}$ = 2

## Appendix A

To prove Theorem 1, the following lemma is needed.

**Lemma** **A1** (**[21] Lemma 1**). *Assume that $x_{i}^{k}$ are non-negative real numbers, i=1,2,⋯,n, and k=1,2,⋯,m. Then,*

$$\sum_{k=1}^{m}{\left(\prod_{i=1}^{n}x_{i}^{k}\right)}^{1/n}\leq \prod_{i=1}^{n}{\left(\sum_{k=1}^{m}x_{i}^{k}\right)}^{1/n}.$$

**Proof of Theorem 1.** By the assumption, all joint probability distributions have a $\left(t_{3}-1\right)$-local decomposition form satisfying Equations (3) and (4). Firstly, take $i_{1}=\cdots =i_{t_{2}}=j_{1}=\cdots =j_{t_{2}}=0$. Then,

$$I_{0,\cdots ,0,0}=\frac{1}{2^{p_{3}}}\sum_{x_{31},\cdots ,x_{3p_{3}}}\langle A_{0}^{11}A_{0}^{21}\cdots A_{0}^{2p_{2}}A_{x_{31}}^{31}\cdots A_{x_{3p_{3}}}^{3p_{3}}\rangle$$

and

$$I_{0,\cdots ,0,1}=\frac{1}{2^{p_{3}}}\sum_{x_{31},\cdots ,x_{3p_{3}}}{\left(-1\right)}^{x_{31}+\cdots +x_{3p_{3}}}\langle A_{0}^{11}A_{0}^{21}\cdots A_{0}^{2p_{2}}A_{x_{31}}^{31}\cdots A_{x_{3p_{3}}}^{3p_{3}}\rangle .$$

Write

$${\langle A_{x_{11}}^{11}\rangle }_{\lambda_{1},\cdots ,\lambda_{p_{2}}}=\sum_{a_{11}}{\left(-1\right)}^{a_{11}}P\left(a_{11}|x_{11},\lambda_{1},\cdots ,\lambda_{p_{2}}\right),$$

$${\langle A_{x_{21}}^{21}\rangle }_{\lambda_{1},\lambda_{p_{2}+1},\cdots ,\lambda_{p_{2}+l_{21}}}=\sum_{a_{21}}{\left(-1\right)}^{a_{21}}P\left(a_{21}|x_{21},\lambda_{1},\lambda_{p_{2}+1},\cdots ,\lambda_{p_{2}+l_{21}}\right),$$

⋯⋯

$${\langle A_{x_{2p_{2}}}^{2p_{2}}\rangle }_{\lambda_{p_{2}},\lambda_{p_{2}+l_{2(p_{2}-1)}+1},\cdots ,\lambda_{t_{3}-1}}=\sum_{a_{2p_{2}}}{\left(-1\right)}^{a_{2p_{2}}}P\left(a_{2p_{2}}|x_{2p_{2}},\lambda_{p_{2}},\lambda_{p_{2}+l_{2(p_{2}-1)}+1},\cdots ,\lambda_{t_{3}-1}\right),$$

$${\langle A_{x_{3j}}^{3j}\rangle }_{\lambda_{p_{2}+j}}=\sum_{a_{3j}}{\left(-1\right)}^{a_{3j}}P\left(a_{3j}|x_{3j},\lambda_{p_{2}+j}\right),\;\;\forall j=1,\cdots ,p_{3}.$$

By Equations (3) and (4) and the facts that $|{\langle A_{0}^{11}\rangle }_{\lambda_{1},\cdots ,\lambda_{p_{2}}}|\leq 1$, $|{\langle A_{0}^{21}\rangle }_{\lambda_{1},\lambda_{p_{2}+1},\cdots ,\lambda_{p_{2}+l_{21}}}|\leq 1$, ⋯, $|{\langle A_{0}^{2p_{2}}\rangle }_{\lambda_{p_{2}},\lambda_{p_{2}+l_{2(p_{2}-1)}+1},\cdots ,\lambda_{t_{3}-1}}|\leq 1$, we have

$$\begin{array}{cc} |I_{0,\cdots ,0,0}|= & \frac{1}{2^{p_{3}}}|\int \cdots \int P_{1}\left(\lambda_{1}\right)\cdots P_{t_{3}-1}\left(\lambda_{t_{3}-1}\right){\langle A_{0}^{11}\rangle }_{\lambda_{1},\cdots ,\lambda_{p_{2}}}{\langle A_{0}^{21}\rangle }_{\lambda_{1},\lambda_{p_{2}+1},\cdots ,\lambda_{p_{2}+l_{21}}}\cdots \\ & \cdot {\langle A_{0}^{2p_{2}}\rangle }_{\lambda_{p_{2}},\lambda_{p_{2}+l_{2(p_{2}-1)}+1},\cdots ,\lambda_{t_{3}-1}}\prod_{j=1}^{p_{3}}\left({\langle A_{0}^{3j}\rangle }_{\lambda_{p_{2}+j}}+{\langle A_{1}^{3j}\rangle }_{\lambda_{p_{2}+j}}\right)d\lambda_{1}\cdots d\lambda_{t_{3}-1}|\\ \leq & \frac{1}{2^{p_{3}}}\int \cdots \int P_{1}\left(\lambda_{1}\right)\cdots P_{t_{3}-1}\left(\lambda_{t_{3}-1}\right)|{\langle A_{0}^{11}\rangle }_{\lambda_{1},\cdots ,\lambda_{p_{2}}}\left|\right|{\langle A_{0}^{21}\rangle }_{\lambda_{1},\lambda_{p_{2}+1},\cdots ,\lambda_{p_{2}+l_{21}}}|\cdots \\ & \cdot |{\langle A_{0}^{2p_{2}}\rangle }_{\lambda_{p_{2}},\lambda_{p_{2}+l_{2(p_{2}-1)}+1},\cdots ,\lambda_{t_{3}-1}}|\prod_{j=1}^{p_{3}}\left|{\langle A_{0}^{3j}\rangle }_{\lambda_{p_{2}+j}}+{\langle A_{1}^{3j}\rangle }_{\lambda_{p_{2}+j}}\right|d\lambda_{1}\cdots d\lambda_{t_{3}-1}\\ \leq & \frac{1}{2^{p_{3}}}\int \cdots \int P_{1}\left(\lambda_{1}\right)\cdots P_{t_{3}-1}\left(\lambda_{t_{3}-1}\right)\prod_{j=1}^{p_{3}}\left|{\langle A_{0}^{3j}\rangle }_{\lambda_{p_{2}+j}}+{\langle A_{1}^{3j}\rangle }_{\lambda_{p_{2}+j}}\right|d\lambda_{1}\cdots d\lambda_{t_{3}-1}\\ = & \prod_{j=1}^{p_{3}}\frac{\int P_{p_{2}+j}\left(\lambda_{p_{2}+j}\right)\left|{\langle A_{0}^{3j}\rangle }_{\lambda_{p_{2}+j}}+{\langle A_{1}^{3j}\rangle }_{\lambda_{p_{2}+j}}\right|d\lambda_{p_{2}+j}}{2}. \end{array}$$

Similarly, one has

$$|I_{0,\cdots ,0,1}|\leq \prod_{j=1}^{p_{3}}\frac{\int P_{p_{2}+j}\left(\lambda_{p_{2}+j}\right)\left|{\langle A_{0}^{3j}\rangle }_{\lambda_{p_{2}+j}}-{\langle A_{1}^{3j}\rangle }_{\lambda_{p_{2}+j}}\right|d\lambda_{p_{2}+j}}{2}.$$

By Lemma A1, we can obtain

$$\begin{array}{cc} & |I_{0,\cdots ,0,0}{|}^{\frac{1}{p_{3}}}+{\left|I_{0,\cdots ,0,1}\right|}^{\frac{1}{p_{3}}}\\ \leq & \prod_{j=1}^{p_{3}}{\left[\int P_{p_{2}+j}\left(\lambda_{p_{2}+j}\right)\left(\frac{|{\langle A_{0}^{3j}\rangle }_{\lambda_{p_{2}+j}}+{\langle A_{1}^{3j}\rangle }_{\lambda_{p_{2}+j}}|}{2}+\frac{|{\langle A_{0}^{3j}\rangle }_{\lambda_{p_{2}+j}}-{\langle A_{1}^{3j}\rangle }_{\lambda_{p_{2}+j}}|}{2}\right)d\lambda_{p_{2}+j}\right]}^{\frac{1}{p_{3}}}\\ = & \prod_{j=1}^{p_{3}}[\int P_{p_{2}+j}\left(\lambda_{p_{2}+j}\right)\max\{|{\langle A_{0}^{3j}\rangle }_{\lambda_{p_{2}+j}}\left|,\right|{\langle A_{1}^{3j}\rangle }_{\lambda_{p_{2}+j}}|\}d\lambda_{p_{2}+j}{]}^{\frac{1}{p_{3}}}\leq 1. \end{array}$$

With similar discussions, Inequality (5) also holds for any other values of $i_{1}$, ⋯, $i_{t_{2}}$, $j_{1}$, ⋯, $j_{t_{2}}\left(\in \left\{0,1\right\}\right)$. □

**Proof of Theorem 2.** For any $i_{1},\cdots ,i_{t_{2}},j_{1},\cdots ,j_{t_{2}}\in \left\{0,1\right\}$, we can obtain

$$I_{i_{1},\cdots ,i_{t_{2}},0}=\frac{1}{2^{p_{3}}}\langle A_{i_{1}}^{11}A_{i_{2}}^{21}\cdots A_{i_{t_{2}}}^{2p_{2}}\left(A_{0}^{31}+A_{1}^{31}\right)\cdots \left(A_{0}^{3p_{3}}+A_{1}^{3p_{3}}\right)\rangle$$

and

$$I_{j_{1},\cdots ,j_{t_{2}},1}=\frac{1}{2^{p_{3}}}\langle A_{j_{1}}^{11}A_{j_{2}}^{21}\cdots A_{j_{t_{2}}}^{2p_{2}}\left(A_{0}^{31}-A_{1}^{31}\right)\cdots \left(A_{0}^{3p_{3}}-A_{1}^{3p_{3}}\right)\rangle .$$

Here, we use similar symbols as those in the proof of Theorem 1. Note that $|\langle A_{i_{1}\left(j_{1}\right)}^{11}\rangle$${}_{\lambda_{1},\cdots ,\lambda_{p_{2}}}|\leq 1$, $|{\langle A_{i_{2}\left(j_{2}\right)}^{21}\rangle }_{\lambda_{1},\lambda_{p_{2}+1},\cdots ,\lambda_{p_{2}+l_{21}}}|\leq 1$, ⋯, $|{\langle A_{i_{t_{2}}\left(j_{t_{2}}\right)}^{2p_{2}}\rangle }_{\lambda_{p_{2}},\lambda_{p_{2}+l_{2(p_{2}-1)}+1},\cdots ,\lambda_{t_{3}-1}}|\leq 1$. By Equation (3) and a similar discussion to that in the proof of Theorem 1, we have

$$|I_{i_{1},\cdots ,i_{t_{2}},0}|\leq \frac{1}{2^{p_{3}}}\int \cdots \int P\left(\lambda_{1},\cdots ,\lambda_{t_{3}-1}\right)\prod_{j=1}^{p_{3}}\left|{\langle A_{0}^{3j}\rangle }_{\lambda_{p_{2}+j}}+{\langle A_{1}^{3j}\rangle }_{\lambda_{p_{2}+j}}\right|d\lambda_{1}\cdots d\lambda_{t_{3}-1}$$

and

$$|I_{j_{1},\cdots ,j_{t_{2}},1}|\leq \frac{1}{2^{p_{3}}}\int \cdots \int P\left(\lambda_{1},\cdots ,\lambda_{t_{3}-1}\right)\prod_{j=1}^{p_{3}}\left|{\langle A_{0}^{3j}\rangle }_{\lambda_{p_{2}+j}}-{\langle A_{1}^{3j}\rangle }_{\lambda_{p_{2}+j}}\right|d\lambda_{1}\cdots d\lambda_{t_{3}-1}.$$

Consequently,

$$\begin{array}{cc} & |I_{i_{1},\cdots ,i_{t_{2}},0}\left|+\right|I_{j_{1},\cdots ,j_{t_{2}},1}|\\ \leq & \frac{1}{2^{p_{3}}}\int \cdots \int P\left(\lambda_{1},\cdots ,\lambda_{t_{3}-1}\right)\prod_{j=1}^{p_{3}}\left(\right|{\langle A_{0}^{3j}\rangle }_{\lambda_{p_{2}+j}}+{\langle A_{1}^{3j}\rangle }_{\lambda_{p_{2}+j}}\left|+\right|{\langle A_{0}^{3j}\rangle }_{\lambda_{p_{2}+j}}-{\langle A_{1}^{3j}\rangle }_{\lambda_{p_{2}+j}}\left|\right)\\ & \cdot d\lambda_{1}\cdots d\lambda_{t_{3}-1}\\ = & \int \cdots \int P\left(\lambda_{1},\cdots ,\lambda_{t_{3}-1}\right)\prod_{j=1}^{p_{3}}\max\{|{\langle A_{0}^{3j}\rangle }_{\lambda_{p_{2}+j}}\left|,\right|{\langle A_{1}^{3j}\rangle }_{\lambda_{p_{2}+j}}\left|\right\}d\lambda_{1}\cdots d\lambda_{t_{3}-1}\leq 1. \end{array}$$

□

**Proof of Equation (8).** Note that

$$\begin{array}{cc} S_{(t_{3}-1)-\mathrm{local}}= & |I_{0,\cdots ,0,0}{|}^{\frac{1}{p_{3}}}+{\left|I_{1,\cdots ,1,1}\right|}^{\frac{1}{p_{3}}}\\ = & \frac{1}{2}{\left(\tau_{1}^{A^{11}A^{21}}\cdots \tau_{1}^{A^{11}A^{2p_{2}}}\tau_{1}^{A^{21}A^{31}}\cdots \tau_{1}^{A^{2p_{2}}A^{3p_{3}}}\right)}^{\frac{1}{2p_{3}}}{\left|\prod_{j=1}^{p_{3}}\left(\cos\beta_{j}+\cos\beta_{j}^{\prime }\right)\right|}^{\frac{1}{p_{3}}}\\ & +\frac{1}{2}{\left(\tau_{2}^{A^{11}A^{21}}\cdots \tau_{2}^{A^{11}A^{2p_{2}}}\tau_{2}^{A^{21}A^{31}}\cdots \tau_{2}^{A^{2p_{2}}A^{3p_{3}}}\right)}^{\frac{1}{2p_{3}}}|\prod_{j=1}^{p_{3}}{\left(\sin\beta_{j}-\sin\beta_{j}^{\prime }\right|}^{\frac{1}{p_{3}}}. \end{array}$$

For convenience, write $f\left(\beta_{1},\beta_{1}^{\prime },\cdots ,\beta_{p_{3}},\beta_{p_{3}}^{\prime }\right)=S_{(t_{3}-1)-\mathrm{local}}$. To maximize the function *f*, calculating all the partial derivatives $\partial_{\beta_{j}}f=\partial_{\beta_{j}^{\prime }}f=0$ for $j,j^{\prime }\in \left\{1,2,\cdots ,p_{3}\right\}$, one obtains that the extreme points of *f* must satisfy

$$\beta_{j}^{\prime }=-\beta_{j}\;\;\mathrm{and}\;\;\left|\tan\beta_{j}\right|=\frac{{\left(\tau_{2}^{A^{11}A^{21}}\cdots \tau_{2}^{A^{11}A^{2p_{2}}}\tau_{2}^{A^{21}A^{31}}\cdots \tau_{2}^{A^{2p_{2}}A^{3p_{3}}}\right)}^{\frac{1}{2p_{3}}}}{{\left(\tau_{1}^{A^{11}A^{21}}\cdots \tau_{1}^{A^{11}A^{2p_{2}}}\tau_{1}^{A^{21}A^{31}}\cdots \tau_{1}^{A^{2p_{2}}A^{3p_{3}}}\right)}^{\frac{1}{2p_{3}}}},\;\;\forall j=1,\cdots ,p_{3}.$$

These imply

$$|\cos\beta_{j}|=\sqrt{\frac{{\left(\tau_{1}^{A^{11}A^{21}}\cdots \tau_{1}^{A^{11}A^{2p_{2}}}\tau_{1}^{A^{21}A^{31}}\cdots \tau_{1}^{A^{2p_{2}}A^{3p_{3}}}\right)}^{\frac{1}{p_{3}}}}{\sum_{i=1}^{2}{\left(\tau_{i}^{A^{11}A^{21}}\cdots \tau_{i}^{A^{11}A^{2p_{2}}}\tau_{i}^{A^{21}A^{31}}\cdots \tau_{i}^{A^{2p_{2}}A^{3p_{3}}}\right)}^{\frac{1}{p_{3}}}}}$$

and

$$|\sin\beta_{j}|=\sqrt{\frac{{\left(\tau_{2}^{A^{11}A^{21}}\cdots \tau_{2}^{A^{11}A^{2p_{2}}}\tau_{2}^{A^{21}A^{31}}\cdots \tau_{2}^{A^{2p_{2}}A^{3p_{3}}}\right)}^{\frac{1}{p_{3}}}}{\sum_{i=1}^{2}{\left(\tau_{i}^{A^{11}A^{21}}\cdots \tau_{i}^{A^{11}A^{2p_{2}}}\tau_{i}^{A^{21}A^{31}}\cdots \tau_{i}^{A^{2p_{2}}A^{3p_{3}}}\right)}^{\frac{1}{p_{3}}}}},\;\forall j=1,\cdots ,p_{3}.$$

Hence, the corresponding function value of these extreme points is

$$\sqrt{\sum_{i=1}^{2}{\left(\tau_{i}^{A^{11}A^{21}}\cdots \tau_{i}^{A^{11}A^{2p_{2}}}\tau_{i}^{A^{21}A^{31}}\cdots \tau_{i}^{A^{2p_{2}}A^{3p_{3}}}\right)}^{\frac{1}{p_{3}}}}.$$

By comparing this function value with those of all endpoints, it follows that the maximum of $f=S_{(t_{3}-1)-\mathrm{local}}$ is

$$S_{(t_{3}-1)-\mathrm{local}}^{\max}=\sqrt{\sum_{i=1}^{2}{\left(\tau_{i}^{A^{11}A^{21}}\cdots \tau_{i}^{A^{11}A^{2p_{2}}}\tau_{i}^{A^{21}A^{31}}\cdots \tau_{i}^{A^{2p_{2}}A^{3p_{3}}}\right)}^{\frac{1}{p_{3}}}}.$$

□

**Proof of Theorem 3.** Assume that Equation (1) holds. Write

$${\langle A_{x_{11}}^{11}\rangle }_{\lambda_{1},\cdots ,\lambda_{p_{2}}}=\sum_{a_{11}}{\left(-1\right)}^{a_{11}}P\left(a_{11}|x_{11},\lambda_{1},\cdots ,\lambda_{p_{2}}\right),$$

⋯⋯

$$\begin{array}{cc} & {\langle A_{x_{\left(n-1\right)p_{n-1}}}^{\left(n-1\right)p_{n-1}}\rangle }_{\lambda_{t_{n-1}-1},\lambda_{t_{n-1}+l_{\left(n-1\right)\left(p_{n-1}-1\right)}},\cdots ,\lambda_{t_{n}-1}}\\ = & \sum_{a_{\left(n-1\right)p_{n-1}}}{\left(-1\right)}^{a_{\left(n-1\right)p_{n-1}}}P\left(a_{\left(n-1\right)p_{n-1}}|x_{\left(n-1\right)p_{n-1}},\lambda_{t_{n-1}-1},\lambda_{t_{n-1}+l_{\left(n-1\right)\left(p_{n-1}-1\right)}},\cdots ,\lambda_{t_{n}-1}\right), \end{array}$$

$${\langle A_{x_{nk}}^{nk}\rangle }_{\lambda_{t_{n-1}-1+k}}=\sum_{a_{nk}}{\left(-1\right)}^{a_{nk}}P\left(a_{nk}|x_{nk},\lambda_{t_{n-1}-1+k}\right),\;\;\forall k=1,\cdots ,p_{n}.$$

By Equation (1), for any $i_{1},\cdots ,i_{t_{n-1}},j_{1},\cdots ,j_{t_{n-1}}\in \left\{0,1\right\}$, we have

$$\begin{array}{cc} & |I_{i_{1},\cdots ,i_{t_{n-1}},0}\left|=\right|\frac{1}{2^{p_{n}}}\sum_{x_{n1},\cdots ,x_{np_{n}}}\langle A_{i_{1}}^{11}\cdots A_{i_{t_{n-1}}}^{\left(n-1\right)p_{n-1}}A_{x_{n1}}^{n1}\cdots A_{x_{np_{n}}}^{np_{n}}\rangle |\\ = & |\frac{1}{2^{p_{n}}}\sum_{x_{n1},\cdots ,x_{np_{n}}}\sum_{a_{11},\cdots ,a_{np_{n}}}{\left(-1\right)}^{a_{11}+\cdots +a_{np_{n}}}\int \cdots \int P\left(\lambda_{1},\cdots ,\lambda_{t_{n}-1}\right)[P\left(a_{11}|i_{1},\lambda_{1},\cdots ,\lambda_{p_{2}}\right)\cdots \\ & \cdot P\left(a_{\left(n-1\right)p_{n-1}}|i_{t_{n-1}},\lambda_{t_{n-1}-1},\lambda_{t_{n-1}+l_{\left(n-1\right)\left(p_{n-1}-1\right)}},\cdots ,\lambda_{t_{n}-1}\right)P\left(a_{n1}|x_{n1},\lambda_{t_{n-1}}\right)\cdots \\ & \cdot P\left(a_{np_{n}}|x_{np_{n}},\lambda_{t_{n}-1}\right)]d\lambda_{1}\cdots d\lambda_{t_{n}-1}|\\ = & |\frac{1}{2^{p_{n}}}\sum_{x_{n1},\cdots ,x_{np_{n}}}\int \cdots \int P\left(\lambda_{1},\cdots ,\lambda_{t_{n}-1}\right){\langle A_{i_{1}}^{11}\rangle }_{\lambda_{1},\cdots ,\lambda_{p_{2}}}\cdots \\ & \cdot {\langle A_{i_{t_{n-1}}}^{\left(n-1\right)p_{n-1}}\rangle }_{\lambda_{t_{n-1}-1},\lambda_{t_{n-1}+l_{\left(n-1\right)\left(p_{n-1}-1\right)}},\cdots ,\lambda_{t_{n}-1}}{\langle A_{x_{n1}}^{n1}\rangle }_{\lambda_{t_{n-1}}}\cdots {\langle A_{x_{np_{n}}}^{np_{n}}\rangle }_{\lambda_{t_{n}-1}}d\lambda_{1}\cdots d\lambda_{t_{n}-1}|\\ = & |\frac{1}{2^{p_{n}}}\int \cdots \int P\left(\lambda_{1},\cdots ,\lambda_{t_{n}-1}\right){\langle A_{i_{1}}^{11}\rangle }_{\lambda_{1},\cdots ,\lambda_{p_{2}}}\cdots \\ & \cdot {\langle A_{i_{t_{n-1}}}^{\left(n-1\right)p_{n-1}}\rangle }_{\lambda_{t_{n-1}-1},\lambda_{t_{n-1}+l_{\left(n-1\right)\left(p_{n-1}-1\right)}},\cdots ,\lambda_{t_{n}-1}}\left({\langle A_{0}^{n1}\rangle }_{\lambda_{t_{n-1}}}+{\langle A_{1}^{n1}\rangle }_{\lambda_{t_{n-1}}}\right)\cdots \\ & \cdot \left({\langle A_{0}^{np_{n}}\rangle }_{\lambda_{t_{n}-1}}+{\langle A_{1}^{np_{n}}\rangle }_{\lambda_{t_{n}-1}}\right)d\lambda_{1}\cdots d\lambda_{t_{n}-1}|\\ \leq & \frac{1}{2^{p_{n}}}\int \cdots \int P\left(\lambda_{1},\cdots ,\lambda_{t_{n}-1}\right)\left|{\langle A_{i_{1}}^{11}\rangle }_{\lambda_{1},\cdots ,\lambda_{p_{2}}}\right|\cdots \\ & \cdot |{\langle A_{i_{t_{n-1}}}^{\left(n-1\right)p_{n-1}}\rangle }_{\lambda_{t_{n-1}-1},\lambda_{t_{n-1}+l_{\left(n-1\right)\left(p_{n-1}-1\right)}},\cdots ,\lambda_{t_{n}-1}}\left|\right|{\langle A_{0}^{n1}\rangle }_{\lambda_{t_{n-1}}}+{\langle A_{1}^{n1}\rangle }_{\lambda_{t_{n-1}}}|\cdots \\ & \cdot |{\langle A_{0}^{np_{n}}\rangle }_{\lambda_{t_{n}-1}}+{\langle A_{1}^{np_{n}}\rangle }_{\lambda_{t_{n}-1}}|d\lambda_{1}\cdots d\lambda_{t_{n}-1}\\ \leq & \frac{1}{2^{p_{n}}}\int \cdots \int P\left(\lambda_{1},\cdots ,\lambda_{t_{n}-1}\right)|{\langle A_{0}^{n1}\rangle }_{\lambda_{t_{n-1}}}+{\langle A_{1}^{n1}\rangle }_{\lambda_{t_{n-1}}}\left|\cdots \right|{\langle A_{0}^{np_{n}}\rangle }_{\lambda_{t_{n}-1}}+{\langle A_{1}^{np_{n}}\rangle }_{\lambda_{t_{n}-1}}|\\ & \cdot d\lambda_{1}\cdots d\lambda_{t_{n}-1}\\ = & \frac{1}{2^{p_{n}}}\int \cdots \int P\left(\lambda_{1},\cdots ,\lambda_{t_{n}-1}\right)\prod_{k=1}^{p_{n}}\left|{\langle A_{0}^{nk}\rangle }_{\lambda_{t_{n-1}-1+k}}+{\langle A_{1}^{nk}\rangle }_{\lambda_{t_{n-1}-1+k}}\right|d\lambda_{1}\cdots d\lambda_{t_{n}-1} \end{array}$$

and

$$\begin{array}{cc} & |I_{j_{1},\cdots ,j_{t_{n-1}},1}\left|=\right|\frac{1}{2^{p_{n}}}\sum_{x_{n1},\cdots ,x_{np_{n}}}{\left(-1\right)}^{x_{n1}+\cdots +x_{np_{n}}}\langle A_{j_{1}}^{11}\cdots A_{j_{t_{n-1}}}^{\left(n-1\right)p_{n-1}}A_{x_{n1}}^{n1}\cdots A_{x_{np_{n}}}^{np_{n}}\rangle |\\ = & |\frac{1}{2^{p_{n}}}\sum_{x_{n1},\cdots ,x_{np_{n}}}{\left(-1\right)}^{x_{n1}+\cdots +x_{np_{n}}}\sum_{a_{11},\cdots ,a_{np_{n}}}{\left(-1\right)}^{a_{11}+\cdots +a_{np_{n}}}\int \cdots \int P\left(\lambda_{1},\cdots ,\lambda_{t_{n}-1}\right)\\ & \cdot [P\left(a_{11}|j_{1},\lambda_{1},\cdots ,\lambda_{p_{2}}\right)\cdots P\left(a_{\left(n-1\right)p_{n-1}}|j_{t_{n-1}},\lambda_{t_{n-1}-1},\lambda_{t_{n-1}+l_{\left(n-1\right)\left(p_{n-1}-1\right)}},\cdots ,\lambda_{t_{n}-1}\right)\\ & \cdot P\left(a_{n1}|x_{n1},\lambda_{t_{n-1}}\right)\cdots P\left(a_{np_{n}}|x_{np_{n}},\lambda_{t_{n}-1}\right)]d\lambda_{1}\cdots d\lambda_{t_{n}-1}|\\ = & |\frac{1}{2^{p_{n}}}\sum_{x_{n1},\cdots ,x_{np_{n}}}{\left(-1\right)}^{x_{n1}+\cdots +x_{np_{n}}}\int \cdots \int P\left(\lambda_{1},\cdots ,\lambda_{t_{n}-1}\right){\langle A_{j_{1}}^{11}\rangle }_{\lambda_{1},\cdots ,\lambda_{p_{2}}}\cdots \\ & \cdot {\langle A_{j_{t_{n-1}}}^{\left(n-1\right)p_{n-1}}\rangle }_{\lambda_{t_{n-1}-1},\lambda_{t_{n-1}+l_{\left(n-1\right)\left(p_{n-1}-1\right)}},\cdots ,\lambda_{t_{n}-1}}{\langle A_{x_{n1}}^{n1}\rangle }_{\lambda_{t_{n-1}}}\cdots {\langle A_{x_{np_{n}}}^{np_{n}}\rangle }_{\lambda_{t_{n}-1}}d\lambda_{1}\cdots d\lambda_{t_{n}-1}|\\ = & |\frac{1}{2^{p_{n}}}\int \cdots \int P\left(\lambda_{1},\cdots ,\lambda_{t_{n}-1}\right){\langle A_{j_{1}}^{11}\rangle }_{\lambda_{1},\cdots ,\lambda_{p_{2}}}\cdots \\ & \cdot {\langle A_{j_{t_{n-1}}}^{\left(n-1\right)p_{n-1}}\rangle }_{\lambda_{t_{n-1}-1},\lambda_{t_{n-1}+l_{\left(n-1\right)\left(p_{n-1}-1\right)}},\cdots ,\lambda_{t_{n}-1}}\left({\langle A_{0}^{n1}\rangle }_{\lambda_{t_{n-1}}}-{\langle A_{1}^{n1}\rangle }_{\lambda_{t_{n-1}}}\right)\cdots \\ & \cdot \left({\langle A_{0}^{np_{n}}\rangle }_{\lambda_{t_{n}-1}}-{\langle A_{1}^{np_{n}}\rangle }_{\lambda_{t_{n}-1}}\right)d\lambda_{1}\cdots d\lambda_{t_{n}-1}|\\ \leq & \frac{1}{2^{p_{n}}}\int \cdots \int P\left(\lambda_{1},\cdots ,\lambda_{t_{n}-1}\right)\left|{\langle A_{j_{1}}^{11}\rangle }_{\lambda_{1},\cdots ,\lambda_{p_{2}}}\right|\cdots \\ & \cdot |{\langle A_{j_{t_{n-1}}}^{\left(n-1\right)p_{n-1}}\rangle }_{\lambda_{t_{n-1}-1},\lambda_{t_{n-1}+l_{\left(n-1\right)\left(p_{n-1}-1\right)}},\cdots ,\lambda_{t_{n}-1}}|\\ & \cdot |{\langle A_{0}^{n1}\rangle }_{\lambda_{t_{n-1}}}-{\langle A_{1}^{n1}\rangle }_{\lambda_{t_{n-1}}}\left|\cdots \right|{\langle A_{0}^{np_{n}}\rangle }_{\lambda_{t_{n}-1}}-{\langle A_{1}^{np_{n}}\rangle }_{\lambda_{t_{n}-1}}|d\lambda_{1}\cdots d\lambda_{t_{n}-1}\\ \leq & \frac{1}{2^{p_{n}}}\int \cdots \int P\left(\lambda_{1},\cdots ,\lambda_{t_{n}-1}\right)|{\langle A_{0}^{n1}\rangle }_{\lambda_{t_{n-1}}}-{\langle A_{1}^{n1}\rangle }_{\lambda_{t_{n-1}}}\left|\cdots \right|{\langle A_{0}^{np_{n}}\rangle }_{\lambda_{t_{n}-1}}-{\langle A_{1}^{np_{n}}\rangle }_{\lambda_{t_{n}-1}}|\\ & \cdot d\lambda_{1}\cdots d\lambda_{t_{n}-1}\\ = & \frac{1}{2^{p_{n}}}\int \cdots \int P\left(\lambda_{1},\cdots ,\lambda_{t_{n}-1}\right)\prod_{k=1}^{p_{n}}\left|{\langle A_{0}^{nk}\rangle }_{\lambda_{t_{n-1}-1+k}}-{\langle A_{1}^{nk}\rangle }_{\lambda_{t_{n-1}-1+k}}\right|d\lambda_{1}\cdots d\lambda_{t_{n}-1}. \end{array}$$

Therefore,

$$\begin{array}{cc} & |I_{i_{1},\cdots ,i_{t_{n-1}},0}\left|+\right|I_{j_{1},\cdots ,j_{t_{n-1}},1}|\\ \leq & \frac{1}{2^{p_{n}}}\int \cdots \int P\left(\lambda_{1},\cdots ,\lambda_{t_{n}-1}\right)\prod_{k=1}^{p_{n}}\left(\right|{\langle A_{0}^{nk}\rangle }_{\lambda_{t_{n-1}-1+k}}+{\langle A_{1}^{nk}\rangle }_{\lambda_{t_{n-1}-1+k}}|\\ & +|{\langle A_{0}^{nk}\rangle }_{\lambda_{t_{n-1}-1+k}}-{\langle A_{1}^{nk}\rangle }_{\lambda_{t_{n-1}-1+k}}\left|\right)d\lambda_{1}\cdots d\lambda_{t_{n}-1}\\ = & \int \cdots \int P\left(\lambda_{1},\cdots ,\lambda_{t_{n}-1}\right)\prod_{k=1}^{p_{n}}\max\{|{\langle A_{0}^{nk}\rangle }_{\lambda_{t_{n-1}-1+k}}\left|,\right|{\langle A_{1}^{nk}\rangle }_{\lambda_{t_{n-1}-1+k}}\left|\right\}d\lambda_{1}\cdots d\lambda_{t_{n}-1}\leq 1, \end{array}$$

as desired. Moreover, if Equation (2) also holds, we have

$$\begin{array}{cc} & |I_{i_{1},\cdots ,i_{t_{n-1}},0}|\\ \leq & \frac{1}{2^{p_{n}}}\int \cdots \int P_{1}\left(\lambda_{1}\right)\cdots P_{t_{n}-1}\left(\lambda_{t_{n}-1}\right)\prod_{k=1}^{p_{n}}\left|{\langle A_{0}^{nk}\rangle }_{\lambda_{t_{n-1}-1+k}}+{\langle A_{1}^{nk}\rangle }_{\lambda_{t_{n-1}-1+k}}\right|d\lambda_{1}\cdots d\lambda_{t_{n}-1}\\ = & \prod_{k=1}^{p_{n}}\int P_{t_{n-1}-1+k}\left(\lambda_{t_{n-1}-1+k}\right)\frac{|{\langle A_{0}^{nk}\rangle }_{\lambda_{t_{n-1}-1+k}}+{\langle A_{1}^{nk}\rangle }_{\lambda_{t_{n-1}-1+k}}|}{2}d\lambda_{t_{n-1}-1+k} \end{array}$$

and

$$\begin{array}{cc} & |I_{j_{1},\cdots ,j_{t_{n-1}},1}|\\ \leq & \frac{1}{2^{p_{n}}}\int \cdots \int P_{1}\left(\lambda_{1}\right)\cdots P_{t_{n}-1}\left(\lambda_{t_{n}-1}\right)\prod_{k=1}^{p_{n}}\left|{\langle A_{0}^{nk}\rangle }_{\lambda_{t_{n-1}-1+k}}-{\langle A_{1}^{nk}\rangle }_{\lambda_{t_{n-1}-1+k}}\right|d\lambda_{1}\cdots d\lambda_{t_{n}-1}\\ = & \prod_{k=1}^{p_{n}}\int P_{t_{n-1}-1+k}\left(\lambda_{t_{n-1}-1+k}\right)\frac{|{\langle A_{0}^{nk}\rangle }_{\lambda_{t_{n-1}-1+k}}-{\langle A_{1}^{nk}\rangle }_{\lambda_{t_{n-1}-1+k}}|}{2}d\lambda_{t_{n-1}-1+k}. \end{array}$$

Using Lemma A1, we obtain

$$\begin{array}{cc} & |I_{i_{1},\cdots ,i_{t_{n-1}},0}{|}^{\frac{1}{p_{n}}}+{\left|I_{j_{1},\cdots ,j_{t_{n-1}},1}\right|}^{\frac{1}{p_{n}}}\\ \leq & \prod_{k=1}^{p_{n}}[\int P_{t_{n-1}-1+k}\left(\lambda_{t_{n-1}-1+k}\right)(\frac{|{\langle A_{0}^{nk}\rangle }_{\lambda_{t_{n-1}-1+k}}+{\langle A_{1}^{nk}\rangle }_{\lambda_{t_{n-1}-1+k}}|}{2}\\ & +\frac{|{\langle A_{0}^{nk}\rangle }_{\lambda_{t_{n-1}-1+k}}-{\langle A_{1}^{nk}\rangle }_{\lambda_{t_{n-1}-1+k}}|}{2}{)d\lambda_{t_{n-1}-1+k}]}^{\frac{1}{p_{n}}}\\ = & \prod_{k=1}^{p_{n}}[\int P_{t_{n-1}-1+k}\left(\lambda_{t_{n-1}-1+k}\right)\max\{|{\langle A_{0}^{nk}\rangle }_{\lambda_{t_{n-1}-1+k}}\left|,\right|{\langle A_{1}^{nk}\rangle }_{\lambda_{t_{n-1}-1+k}}|\}d\lambda_{t_{n-1}-1+k}{]}^{\frac{1}{p_{n}}}\leq 1, \end{array}$$

that is, Inequality (9) holds. □
